# Dynamic Covalent Lewis Pair Grafting Enables Spontaneous Linear-to-Bottlebrush Polymer Assembly and Adaptive Elastomers

**DOI:** 10.1021/acsmacrolett.6c00433

**Published:** 2026-10-02

**Authors:** Edip Ajvazi, Verena Schinegger, Milan Kracalik, Oliver Brüggemann, Ingrid Graz, Uwe Monkowius, Ian Teasdale

**Affiliations:** † Institute of Polymer Chemistry, 27266 Johannes Kepler University Linz, Altenberger Straße 69, 4040 Linz, Austria; ‡ Institute of Polymer Science, 27266 Johannes Kepler University Linz, Altenberger Straße 69, 4040 Linz, Austria; § Institute of Inorganic Chemistry, 27266 Johannes Kepler University Linz, Altenberger Straße 69, 4040 Linz, Austria; ∥ School of Education, STEM Education, 27266 Johannes Kepler University Linz, Altenberger Straße 69, 4040 Linz, Austria; ⊥ Institute of Biophysics, Life Science Center, Johannes Kepler Universität Linz, Gruberstrasse 40, 4020 Linz, Austria

## Abstract

Bottlebrush and comb-graft polymers, macromolecules with densely grafted side chains along a central backbone, exhibit distinctive conformational, rheological, and self-assembly behaviors. Although these architectures are widely used to tune mechanical responses, most reported systems rely on stable covalent carbon frameworks, or fragile supramolecular assemblies, while dynamic covalent chemistries remain largely unexplored. Herein, we harness Lewis acid–base pairs whose directional coordinate bonds drive the spontaneous assembly of graft architectures by simply mixing Lewis acid-terminated polydimethylsiloxane (PDMS) oligomers with the polymeric Lewis base poly­(4-vinylpyridine) (P4VP). The PDMS-oligomers efficiently graft to the P4VP backbone. ^11^B and ^1^H NMR studies revealing a dynamic-covalent equilibrium, with rapid kinetics. Upon addition of a difunctional boronate cross-linker, the dynamically grafted polymers form stretchable elastomeric networks that can be thermally reprocessed and undergo autonomous self-healing. This Lewis-pair strategy expands bottlebrush polymer chemistry beyond static covalent and fragile supramolecular designs, with a simple, tunable route to recyclable covalent adaptable networks of soft and elastic materials.

## Graphical abstract

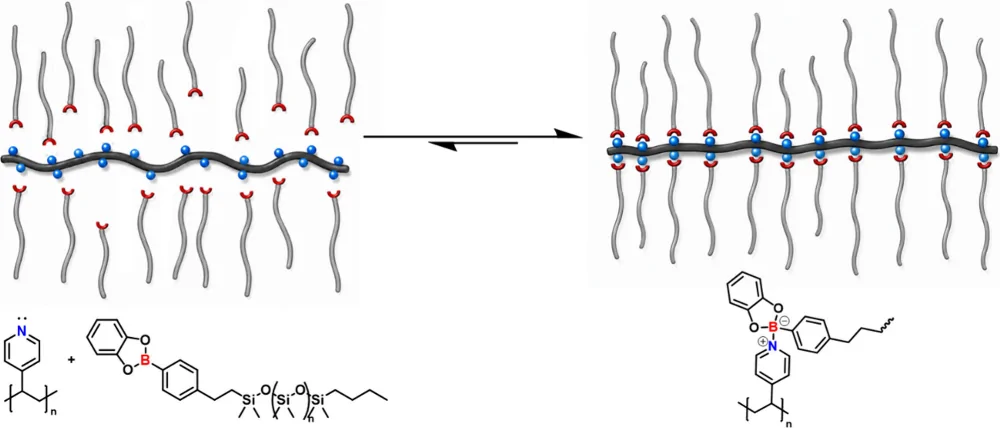

Bottlebrush and comb-graft polymers have emerged as an important class of soft materials because their architecture enables independent control over chain conformation, rheological and mechanical properties.^1–4^ Their properties are primarily governed by the backbone length (N_BB_), side-chain length (N_SC_), grafting density (N_g_), and the chemical functionality of the polymer chains.^1–3,5,6^ Compared with conventional linear polymers, bottlebrush architectures provide an additional level of structural control and a high density of functional chain ends, enabling precise tuning of mechanical, rheological, and interfacial properties.^1–3,5–7^ This unique combination of architectural versatility and functionality has led to applications across polymer and materials science, including organic electronics,^8^ ultrasoft elastomers,^5,6,9,10^ and vibration- or energy-dissipating materials.^2,5^

In parallel, dynamic covalent chemistry has emerged as a powerful strategy for imparting adaptive functionality to polymeric materials, enabling self-healing, reprocessability, and sustainability-driven recyclability without compromising structural integrity.^11^ The integration of dynamic bonding motifs into polymer networks has therefore attracted significant attention as a route toward next-generation adaptive and circular materials.^12–15^ Despite these advances, the incorporation of dynamic bonds directly within the side-chain architecture of bottlebrush polymers remains largely unexplored. To the best of our knowledge, reports to date have focused primarily on dynamically cross-linked bottlebrush elastomer networks rather than intrinsically dynamic bottlebrush macromolecules. Notable examples include dynamic bottlebrush networks reported by Bates and co-workers, in which transesterification enabled self-healing and reprocessing.^16,17^ Fan and co-workers also reported boronic-ester-cross-linked bottlebrush polymers with self-healing and adhesive properties.^18^ These studies highlight the promise of combining bottlebrush architectures with dynamic chemistry, while also underscoring the opportunity to develop bottlebrush polymers in which dynamic functionality is incorporated directly at the molecular level.

A handful of supramolecular bottlebrush systems have also been reported.^19^ However, since they rely on weak noncovalent forces, constructing a supramolecular backbone must overcome steric repulsion from dense side chains. Ordered architectures also require directional interactions, excluding many ionic and most van der Waals contacts. Striking the right balance has been challenging^19^ but has been achieved, for example, via hybrid systems combining covalent and noncovalent bonding^20^ or relatively complex cyclic peptide based systems.^21^

Lewis pairs (LPs) are reversible but have been underused in recent advances in dynamic covalent polymer networks. Their bond is covalent; “dative” only indicates the electron source, so at suitable strengths they function as dynamic covalent chemistry (DCC) with the local environment of the boron playing a considerable role in tuning equilibrium constants and bond strengths. Crucially, substituents allow straightforward tuning of bond equilibrium, potentially enabling wide control over response temperatures and kinetics of dynamic covalent polymer networks (DCPN). The capture/release process is also simpler than common dynamic reactions like transesterification or transamidation. In particular, Jäkle and co-workers demonstrated polymerizable organoborane Lewis acids for transient PDMS networks,^22,23^ while Shaver and co-workers employed frustrated Lewis pairs to prepare self-healing dynamic gels.^24,25^

Building on these advances, we previously developed a straightforward Lewis pair-based polymer platform by combining organoborane-functionalized PDMS with commercially available Lewis base polymers, demonstrating the potential of archetypal nitrogen–borane adducts for dynamic polymer networks.^26^ Here, we harness B–N Lewis acid–base interactions to direct the spontaneous assembly of comb and bottlebrush architectures.

In this study, we employ a deliberately simple molecular design (Scheme 1), wherein vinyl-functionalized boronic ester building blocks are attached to hydride-terminated PDMS via hydrosilylation, yielding monoend-capped oligomers with systematically varied chain lengths (N_sc_). Facile mixing of the boronic ester-functionalized PDMS oligomers with poly­(4-vinylpyridine) (P4VP) induces dynamic-covalent B–N bonding, reversibly grafting the PDMS side chains onto the P4VP backbone (N_BB_) without catalysts or additional reagents and thereby generating comb- and brush-like polymer architectures. In contrast, a control mixture of P4VP and unfunctionalized PDMS underwent macroscopic phase separation, with P4VP precipitating rather than forming a homogeneous material, supporting the notion that boronate–pyridine coordination is required for compatibilization and dynamic grafting (Figure SI 1).

**Scheme 1:**
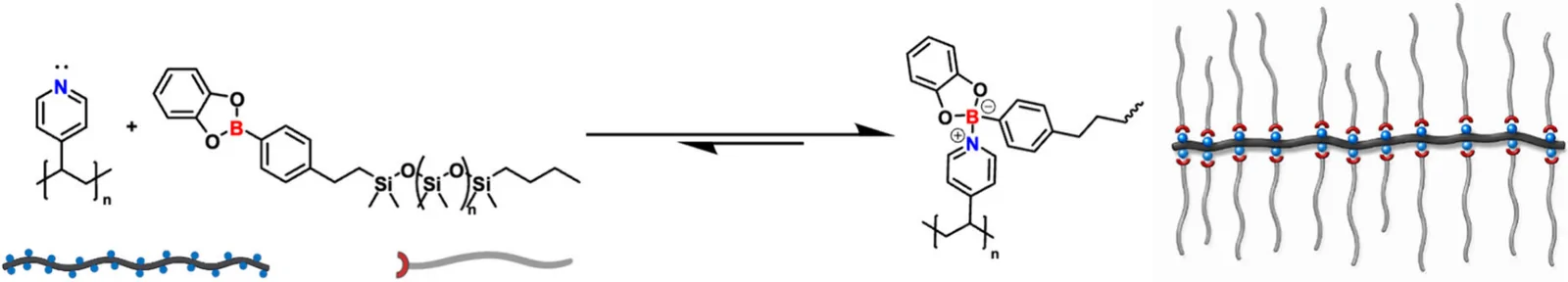
Schematic Representation of the Reversible Lewis Pair Interaction between Boronic Ester-Based Lewis Acids (LA) and Pyridine-Based Lewis Bases (LB) and Its Implementation in Dynamically Associated Polymer Architectures^a^

a The dynamic LA–LB interaction enables reversible connectivity within a simple polymer system.

To probe the dynamic-covalent reaction, small-molecule and macromolecular systems in dichloromethane at room temperature were investigated by ^11^B NMR (Figure 1) and ^1^H NMR spectroscopy (Figures SI 2 and 3). In the small-molecule model study, increasing amounts of 4-ethylpyridine were added to 2-(4-vinylphenyl)­boronic acid catechol ester, resulting in a continuous upfield shift of the population-averaged boron resonance from 32.2 to 11.9 ppm (Figure 1a). This response is consistent with a progressive shift in the equilibrium from the trigonal planar, uncoordinated boronic ester toward the tetrahedral B–N coordinate-covalent adduct. For substoichiometric N/B ratios, the observation of a single population-averaged ^11^B resonance is consistent with fast exchange between uncoordinated and pyridine-coordinated boron environments on the NMR time scale. Using the limiting chemical-shift separation and the coalescence approximation, a lower bound for the exchange rate at room temperature was estimated (see SI).

**Figure 1:**
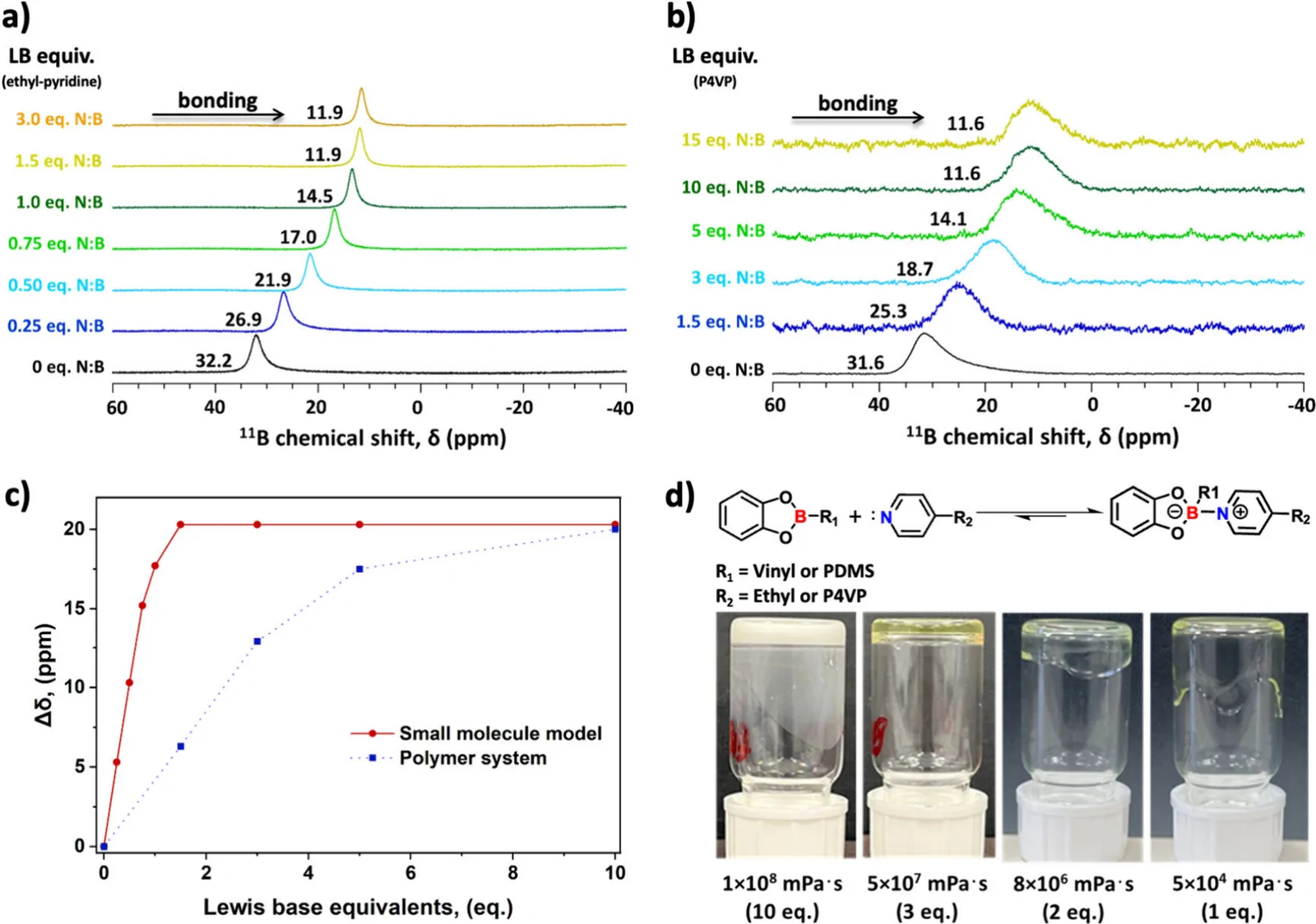
Lewis-pair complexation in molecular and polymeric systems. ^11^B NMR spectra of (a) a small-molecule boronate model and (b) polymer mixtures prepared with increasing Lewis base equivalents, showing an upfield shift upon B–N bonding. (c) Chemical-shift change (Δδ = δ_free_ – δ_obs_), as a function of Lewis-base equivalents, calculated from the signal maxima in the ^11^B NMR spectra. (d) Reaction scheme of the LA–LB adduct formation and vial images of representative Lewis-pair-associated polymer samples. Complex viscosities were evaluated at ω = 0.1 rad·s^–1^ and 25 °C.

The short-chain polymer analogue, B_short_, obtained by dynamically grafting catechol boronate ester-capped PDMS chains (N_sc_ ≈ 14) onto a P4VP (N_BB_ ≈ 1500, Table SI 2) exhibited substantially broader resonances but the same chemical-shift response as the small-molecule study (Figure 1b,c). The broadened, population-averaged signal is consistent with coexisting free and coordinated boron environments within a distribution of local coordination states, as expected for a heterogeneous macromolecular environment with restricted segmental mobility. A distribution of stereochemical environments along the P4VP backbone may also contribute to the line broadening, so the line width is not assigned solely to coordination-state heterogeneity. Whereas the small-molecule model approached its maximum shift at approximately 1–1.5 equiv of Lewis base, the polymer system required approximately 10 equiv of Lewis base to approach a comparable chemical shift. This difference may reflect steric accessibility and the concentration-dependent association equilibrium in the polymer-containing solution. To compare the two systems quantitatively, the chemical-shift change was expressed as Δδ = δ_free_ – δ_obs_, where δ_free_ is the resonance of the free boronate and δ_obs_ is the observed signal maximum at each Lewis-base loading (Figure 1). Accordingly, an increasing positive Δδ reflects the progressive upfield displacement associated with B–N bonding.

The same strategy was applied to longer catechol boronate ester-capped PDMS side chains, B_long_ (N_SC_ ≈ 85). Their lower boron concentration prevented reliable ^11^B NMR analysis. For both the B_short_ and B_long_ series, the nominal side-chain loading, θ_nom_, was calculated as the molar ratio of monofunctional PDMS–boronate chains to 4VP repeat units used in the formulation, expressed as a percentage. The corresponding composition ratio determined by ^1^H NMR integration is denoted θ_NMR_ (Table SI 1 and Figures SI 5–6). Both quantities describe side-chain-to-4VP composition ratios and do not directly quantify the fraction of coordinated side chains. For the samples with nominal side-chain loadings of 10–70%, the NMR-derived values differed from the nominal values by no more than three percentage points.

The formulations display pronounced composition-dependent differences in macroscopic flow behavior, with complex viscosities spanning several orders of magnitude at ω = 0.1 rad·s^–1^ (Figure 1d). These qualitative observations motivated a systematic rheological investigation of the dynamically grafted polymers. Their viscoelastic response is governed by reversible Lewis-pair-mediated intermolecular coupling and depends strongly on the feed ratio of boronate-functionalized PDMS side chains to P4VP, as well as on the resulting polymer architecture. The influence of polymer architecture on the viscoelastic response was investigated by oscillatory shear rheology (Figure 2). The B_short_ series exhibited comparatively high storage moduli and only weak frequency dependence, consistent with strongly coupled, solid-like dynamically associated polymers (Figure 2a). In contrast, the B_long_ formulations showed substantially lower *G*′ values and a more pronounced frequency dependence, indicating enhanced relaxation on the experimental time scale. At comparable nominal side-chain loadings, increasing the PDMS side-chain length reduced the storage modulus by several orders of magnitude. The architecture dependence is further illustrated by the storage modulus evaluated at ω = 0.1 rad·s^–1^ (Figure 2b). For both side-chain lengths, *G*′ decreased with increasing NMR-derived side-chain loading, θ, although the reduction was substantially more pronounced for the B_long_ series. Short PDMS side chains provide limited steric shielding of the P4VP backbone, allowing greater interchain approach and stronger intermolecular coupling. Longer side chains instead favor a more expanded brush-like conformation and increasingly shield the backbone as θ increases, thereby suppressing interpenetration and reducing intermolecular coupling. These results demonstrate that side-chain length is the dominant architectural parameter controlling the translation of dynamic B–N coordination into macroscopic viscoelasticity. Complete frequency sweeps for intermediate nominal side-chain loadings and mixed side-chain compositions are provided in the Supporting Information (Figure SI 8–9). The mixed side-chain formulations exhibited intermediate viscoelastic properties that varied systematically with the ratio of short and long PDMS side chains, further supporting a direct influence of side-chain architecture on the rheological response. Taken together, these results establish qualitative structure–property relationships. Increasing the side-chain loading reduced *G*′ and complex viscosity, particularly for long side chains, while increasing N_SC_ lowered *G*′ by several orders of magnitude and enhanced relaxation. Consistent with this trend, the B_short_ formulations remained predominantly elastic over the experimentally reliable frequency range, whereas the B_long_ series exhibited *G*′/*G*″ crossovers that shifted strongly with side-chain loading (Figure SI 8). This pronounced softening is consistent with enhanced steric shielding and reduced interchain coupling; however, longer PDMS side chains also increase the PDMS mass fraction and decrease the density of coordinating groups per unit mass, which may additionally contribute to the observed response.

**Figure 2:**
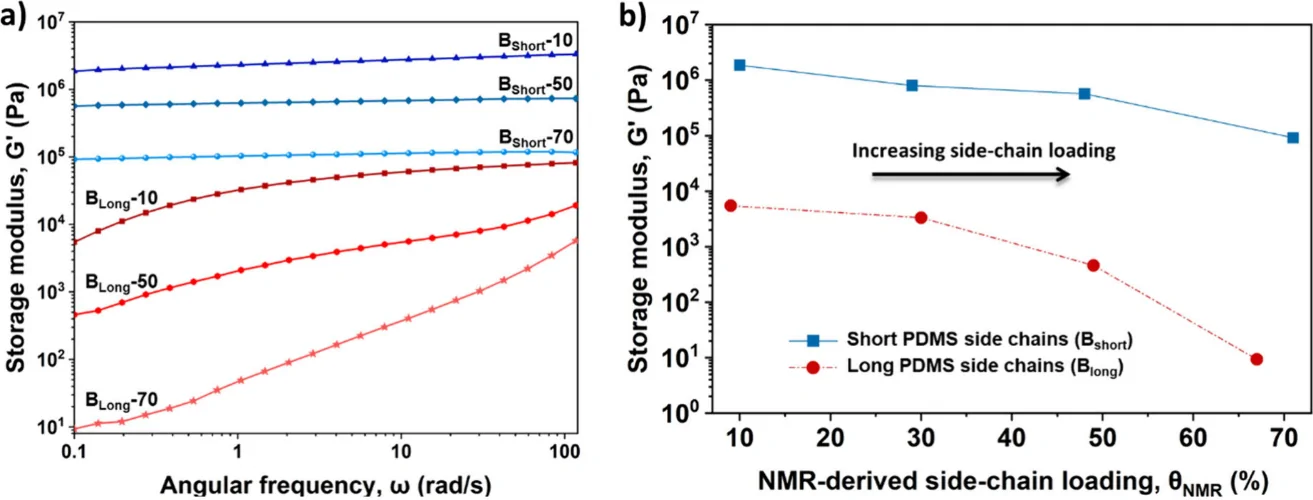
Polymer architecture controls the viscoelastic response of dynamically associated brush-like polymers. (a) Storage modulus (*G*′) as a function of angular frequency for polymers containing short (B_short_, N_sc_ ≈ 14) and long (B_long_, N_sc_ ≈ 85) PDMS side chains. Sample labels indicate the nominal side-chain loading; for example, B_short_-10 denotes θ_nom_ = 10%. (b) Storage modulus evaluated at ω = 0.1 rad s^–1^ as a function of the NMR-derived side-chain loading, θ_NMR_, defined as the molar ratio of monofunctional PDMS–boronate chains to 4VP repeat units determined by ^1^H NMR integration and expressed as a percentage.

To translate the dynamic interactions observed in the grafted polymers into macroscopic materials, elastomeric systems were prepared by introducing difunctional linking units into the system (Figure 3a). The difunctional linker was synthesized based on the same boronic ester motif and subsequently incorporated into the PDMS matrix, enabling the formation of interconnected structures via reversible Lewis pair interactions. The difunctional cross-linker allows the formation of percolated networks, while the cross-links remain dynamic in nature. The composition of the elastomer systems was controlled via the ratio of boronic ester functionalities, Lewis base content, and difunctional linker, allowing systematic variation of cross-linking density and stoichiometry (N/B ratio) (Table 1).

**Table 1:** Compositions of the P4VP/PDMS Elastomer Formulations Calculated from the Amounts of Components Used^a^

elastomer	side-chain loading, θ_nom_ (%)	cross-linker-derived B loading (%)	total N/B ratio	P4VP content (wt%)
A	0	3	33.33	32.1
B	1	3	25.00	26.8
C	0	3.9	25.78	26.8
D	0	50	2.00	2.8
E	1	50	1.96	2.7
F	20	30	2.00	2.9

a The total N/B ratio includes pyridine nitrogen atoms from P4VP and boron atoms from both monofunctional side chains and difunctional crosslinkers. The P4VP content is the calculated mass fraction of P4VP in the total solvent-free formulation, expressed in wt%.

**Figure 3:**
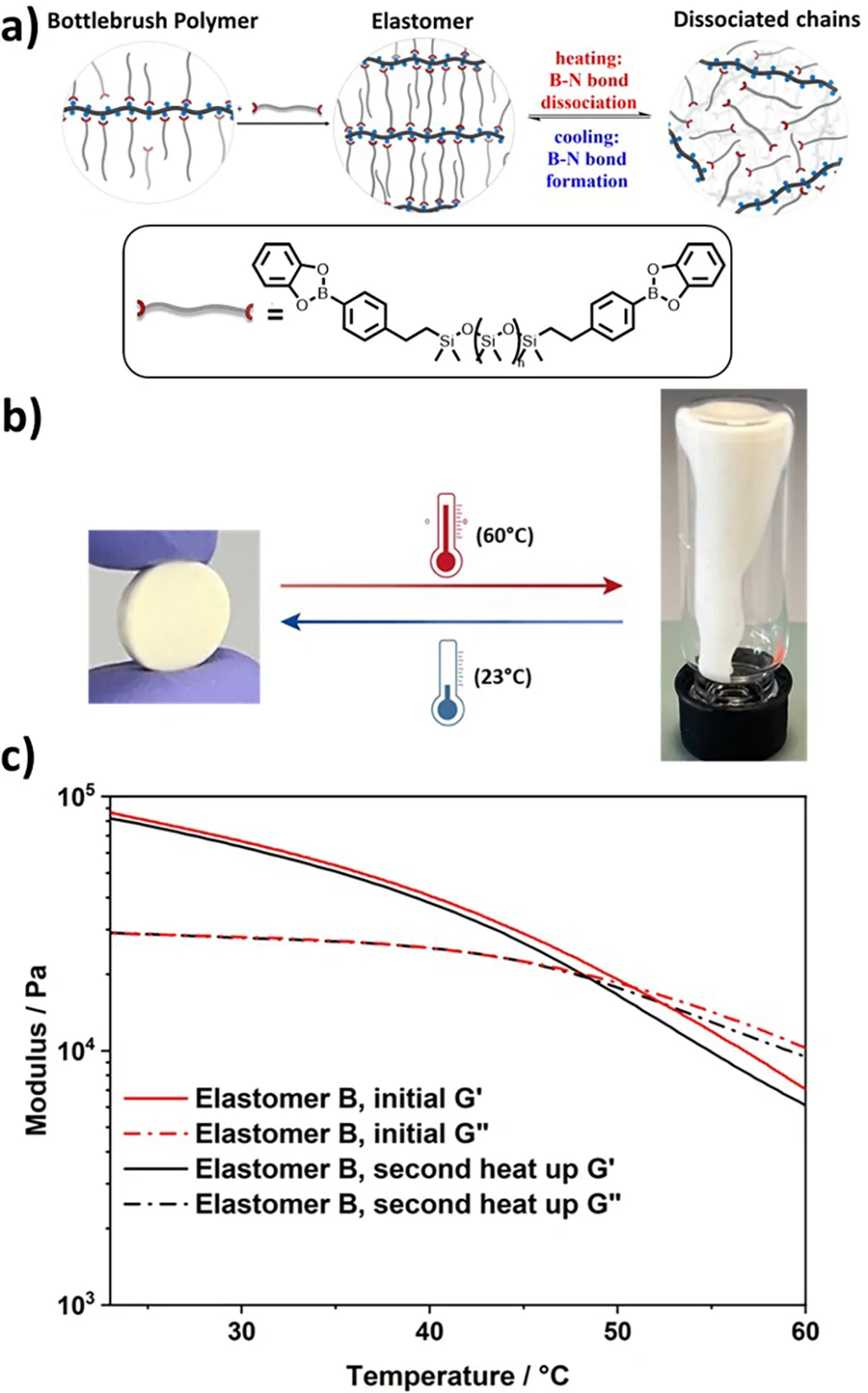
Thermal reversibility of Lewis-pair-cross-linked elastomer B. (a) Schematic illustration of reversible B–N bond dissociation and reformation during heating and cooling. (b) Photographs of elastomer B before and during thermal softening. (c) Temperature-dependent *G*′ and *G*″ recorded during the initial and second heating ramps separated by cooling, showing recovery of the viscoelastic response after one complete thermal cycle.

Elastomeric formulations were prepared using the graft-containing formulation B as the model system. The nominal side-chain loading is defined on the same basis as for the un-cross-linked formulations discussed above. Elastomer B has θ_nom_ = 1%, corresponding to one monofunctional PDMS side chain per 100 4VP repeat units, whereas the formulations investigated in Figure 2 have nominal side-chain loadings of 10–70%. Boronate groups contributed by the difunctional cross-linker are reported separately in Table 1 and are not included in θ_nom_. To distinguish the influence of the graft architecture from changes in composition, formulation B was compared with three reference systems. Elastomer A contained the same nominal cross-linker content but no grafted side chains, thereby isolating the effect of the graft architecture. Elastomer C contained no monofunctional PDMS side chains; instead, the amount of difunctional PDMS linker was increased from 3.0% to 3.9% cross-linker-derived B loading to match the total mass of PDMS-based components in formulation B, as detailed in the Supporting Information. P4VP was characterized separately to assess the contribution of the Lewis-base polymer to the viscoelastic response. The rheological response clearly highlights the impact of the graft architecture. While formulations A and C, which contain no grafted side chains, exhibited comparatively high storage moduli with only weak frequency dependence, formulation B showed a substantially reduced *G*′ together with more pronounced frequency dependence, indicating enhanced stress relaxation. This behavior was accompanied by a lower complex viscosity and higher tan δ, consistent with increased energy dissipation (Figure SI 10). P4VP plays a dual role by providing the Lewis-base sites required for reversible B–N coordination while simultaneously contributing to the overall stiffness owing to its comparatively high glass-transition temperature. The transition from a transparent to an opaque appearance with increasing P4VP content further suggests the development of microstructural heterogeneity. To further probe the influence of composition, a second elastomer series with reduced P4VP content (D–F) was investigated (Figure SI 11). The same architectural trend was retained, with graft-containing formulations exhibiting lower *G*′ and higher tan δ, although the differences between the corresponding control and graft-containing formulations were less pronounced than in the first series. This behavior likely reflects the combined effects of the substantially lower P4VP content, the correspondingly reduced Lewis-base-to-boron ratio, and the higher PDMS fraction, which together modify the contribution of reversible B–N coordination to the overall viscoelastic response. Stress-relaxation experiments further support this interpretation (Figure SI 12). At low cross-linker content, formulation B relaxed substantially faster than A, demonstrating that the graft architecture promotes network rearrangement under these conditions. In contrast, the high-cross-link formulations (D and F) exhibited more similar relaxation behavior, indicating that the influence of the graft architecture becomes less pronounced in the second formulation series. The elastomer formulations exhibited low-frequency storage moduli in the low-kilopascal range, placing them within the supersoft regime described for bottlebrush polymer networks.^2,6,10^

The thermoreversible nature of the dynamically grafted elastomers was first demonstrated macroscopically using formulation B (Figure 3a,b). Formulation B has θ_nom_ = 1% and is therefore described here as a sparsely grafted comb-like network. Upon heating, the initially self-supporting elastomer gradually softened and flowed under its own weight, consistent with increased chain mobility and a temperature-dependent shift in reversible B–N association. Cooling restored a solid, self-supporting material, indicating reformation of the reversible Lewis-pair bonds. The macroscopic observations are supported by temperature-dependent rheological measurements (Figure 3c). Both *G*′ and *G*″ decreased continuously with increasing temperature, reflecting progressive bond dissociation and enhanced chain mobility. After the first heating ramp and subsequent cooling, a second heating ramp was recorded, and the viscoelastic response was largely recovered, with only minor deviations between the two ramps. The similar storage and loss moduli support recovery of the viscoelastic state after one complete thermal cycle. These results confirm the thermoreversible character of the dynamically grafted elastomer and highlight its potential for thermal reprocessing and recycling. Additional rheological characterization, including amplitude sweeps, step-strain recovery, temperature-dependent complex viscosity, and tan δ measurements, is provided in the Supporting Information (Figures SI 13 and 15). The viscoelastic response of elastomer F was also largely retained after a heating cycle (Figure SI 14b). Formulation F has the highest nominal side-chain loading in the elastomer series (θ_nom_ = 20%) and is the closest of these formulations to the bottlebrush-like regime. Because F is very soft and difficult to handle reproducibly as a tensile specimen, it was not selected for the main-text mechanical demonstrations. The rheological and thermal behavior of F are presented in Figures SI 11–14.

The autonomous healing ability of elastomer B was evaluated using two specimen geometries adapted to the respective mechanical tests (Figure 4). Strip-shaped specimens were used for tensile characterization, whereas disc-shaped specimens were employed for oscillatory rheology. After cutting, the corresponding specimen halves were brought into contact and allowed to heal for 24 h under ambient conditions without external stimulation. The healed tensile strips retained pronounced extensibility (Figure 4a) and exhibited a stress–strain response closely resembling that of the pristine material (Figure 4b). Only minor differences in maximum stress were observed, while the characteristic deformation behavior was largely preserved. The higher stress measured for the pristine specimen at 1000 mm·min^–1^ relative to 40 mm·min^–1^ illustrates the pronounced rate dependence of the tensile response (Figure 4b). Independently prepared disc-shaped specimens were used to evaluate recovery of the linear viscoelastic response (Figure 4c). The nearly overlapping frequency-dependent *G*′ and *G*″ values of the pristine and healed discs indicate efficient restoration of the dynamic network connectivity. Together, these experiments demonstrate autonomous healing across different specimen geometries while largely preserving both tensile and rheological properties.

**Figure 4:**
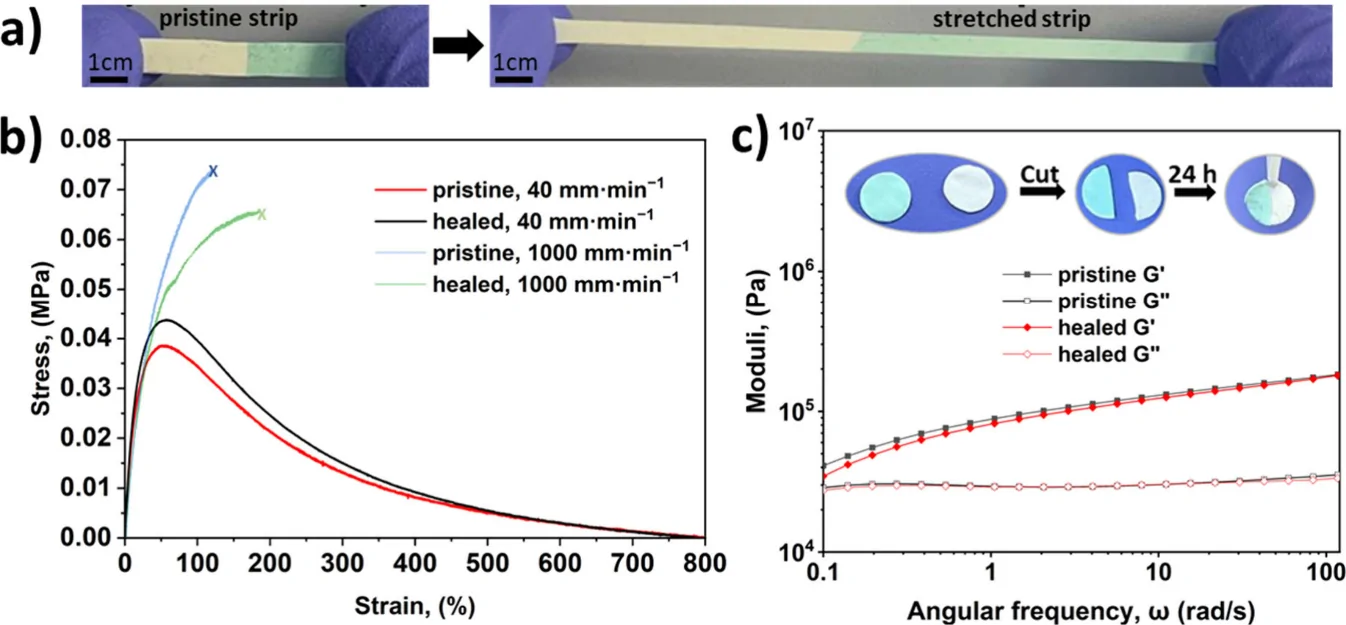
Mechanical performance and autonomous self-healing of elastomer B. (a) Photographs of the pristine elastomer strip before and during tensile deformation. (b) Stress–strain curves of the pristine and healed elastomer at 40 mm·min^–1^, together with a pristine and healed curve at 1000 mm·min^–1^. (c) Frequency-dependent storage and loss moduli of the pristine and healed elastomer. Insets illustrate autonomous healing after cutting and rejoining the specimen for 24 h at ambient conditions.

In conclusion, this study introduces a simple Lewis pair strategy that drives a spontaneous association of boronate-terminated PDMS side chains with P4VP and a composition-dependent progression from sparse comb-like to more densely grafted brush-like architectures. By varying side-chain length and the density of reversible interactions, we identify regimes where architecture or coordination dominates material response, enabling predictive control over flow, relaxation, and modulus. Upon cross-linking, these brushes yield supersoft, highly ductile and strongly rate-dependent elastomers that are thermoreversible, reprocessable, and autonomously self-healing. As the functionalization of polymers by Lewis-acid–base chemistry could in the future be easily expanded to other side-chain polymers, this represents a broadly applicable approach. The introduction of dynamic side-chains expands comb-graft and bottlebrush polymer chemistry beyond static covalent or fragile supramolecular designs and provides a modular platform for covalent adaptable networks and should further extend performance and application space in soft robotics, damping, and recyclable elastomer technologies.

## Supplementary Material



## Notes

Materials and methods, synthetic procedures, NMR spectra and composition analysis, and additional rheological and mechanical characterization (PDF)
