# Supplementary material for: Dynamic Covalent Lewis Pair Grafting Enables Spontaneous Linear-to-Bottlebrush Polymer Assembly and Adaptive Elastomers
Source: ACS Macro Lett. 2026 Oct 2;15(10):1565–71. doi: 10.1021/acsmacrolett.6c00433 (PMC13689928; doi:10.1021/acsmacrolett.6c00433)
Supplement: Supplementary file 1 [file mz6c00433_si_001.pdf]

Supporting Information

Dynamic Covalent Lewis Pair Grafting Enables Spontaneous Linear-to-Bottlebrush Polymer Assembly and Adaptive Elastomers

*Edip Ajvazi[1], Verena Schinegger[1,4], Milan Kracalik[2], Oliver Brüggemann[1], Ingrid Graz[4,5], Uwe Monkowius[3,4] and Ian Teasdale[1]**

[1] Institute of Polymer Chemistry, Johannes Kepler University Linz, Altenberger Straße 69, 4040 Linz, Austria

[2] Institute of Polymer Science, Johannes Kepler University Linz, Altenberger Straße 69, 4040 Linz, Austria

[3] Institute of Inorganic Chemistry, Johannes Kepler University Linz, Altenberger Straße 69, 4040 Linz, Austria

[4] School of Education, STEM Education, Johannes Kepler University Linz, Altenberger Straße 69, 4040 Linz, Austria.

[5] Institute of Biophysics, Life Science Center, Johannes Kepler Universität Linz, Gruberstrasse 40, 4020 Linz, Austria

* ian.teasdale@jku.at

Materials and Methods

Unless otherwise stated, all reactions were carried out under an argon atmosphere using standard Schlenk techniques or in an MBraun UNILab LMF glovebox. All chemicals were purchased from commercial suppliers and used as received unless otherwise noted. Poly(4-vinylpyridine) (P4VP; product 472352) was obtained from Sigma-Aldrich with a supplier-reported weight-average molar mass of Mw ≈ 160,000 g mol^{-1}. Hydride-terminated polydimethylsiloxane (DMS-H25, Mn ≈ 14,400 g mol^{-1}) and monohydride-terminated poly(dimethylsiloxane) (MCR-H21, Mn ≈ 6,300 g mol^{-1}; MCR-H07, Mn ≈ 1,000 g mol^{-1}) were obtained from Gelest. These values are supplier-reported number-average molar masses; the estimated molar masses of the boronate-functionalized derivatives are summarized in Table SI 2. Additional reagents included 4-ethylpyridine (Sigma-Aldrich), catechol, 4-vinylphenylboronic acid (BLD), and Karstedt catalyst (2 wt% in xylene, Sigma-Aldrich).

Toluene, dichloromethane (DCM), acetonitrile, n-heptane, and other solvents were purchased from VWR and used as received. ^{1}H and ^{11}B NMR spectra were recorded on a Bruker Avance 300 spectrometer operating at 300 MHz for ^{1}H and 96 MHz for ^{11}B. Quartz NMR tubes were used for ^{11}B measurements. Chemical shifts were referenced to residual solvent resonances.

Rheological measurements were performed on an Anton Paar MCR 501 rheometer equipped with parallel-plate geometry (10 mm diameter). Samples were prepared as disks with a diameter of 10 mm and a thickness of approximately 1–2 mm. The linear viscoelastic region (LVE) was determined by amplitude sweeps at an angular frequency of 1 $rad \cdot s^{-1}$ over a strain range of 1–200% (Figure SI 15a). Frequency sweeps were conducted at a strain within the LVE (γ = 1%) over an angular frequency range of 0.01–628 $rad \cdot s^{-1}$. Temperature sweeps were performed at γ = 1% and ω = 1 $rad \cdot s^{-1}$ using a heating rate of 1.5 °C min^{-1}. Cyclic step-strain measurements were performed at an angular frequency of ω=1 $rad \cdot s^{-1}$ by alternating between low-strain (γ=1%) and high-strain (γ=300%) conditions for four consecutive cycles to evaluate viscoelastic recovery. Stress-relaxation experiments were performed at room temperature using a strain of 1%, ensuring operation within the LVE. Tensile tests were conducted under ambient conditions using the parameters specified in the corresponding figure captions.

Uniaxial tensile tests were performed under ambient conditions using a Zwick/Roell Z005 universal testing machine equipped with a 2.5 kN load cell. Rectangular specimens of formulation B (10.0 mm × 40.0 mm × 2.0 mm) were tested at crosshead speeds of 40 and 1000 mm min^{-1}. For healed specimens, the cut surfaces were brought into contact without external pressure, and the specimens were stored for 24 h under ambient conditions before testing.

Small-Molecule NMR Titration

(4-Vinylphenyl)boronic acid catechol ester (15.0 mg, 0.068 mmol, 1eq.) was dissolved in 0.6 mL DCM-d_2. 4-Ethylpyridine (0.25, 0.50, 0.75, 1.00, 1.50, 3.00, 5.00 and 10.00 eq.) was added to the LA solution via microlitre syringe and NMR spectra were recorded after each LB addition. The titration was performed sequentially in the same NMR tube. The initial Lewis acid concentration was 0.113 mol L^{-1}. At the final titration point of 10.0 equiv. of 4-ethylpyridine, the total sample volume was approximately 0.677 mL, corresponding to final LA and LB concentrations of 0.100 and 1.00 $mol \cdot L^{-1}$, respectively.

Polymer NMR Study

P4VP (30.0 mg, corresponding to 0.285 mmol of pyridine repeat units) and B_{short} were dissolved in CD_2Cl_2 at N/B ratios of 1.5, 3, 5, 10, and 15. Based on the estimated molar mass of B_{short} (Mn ≈ 1.2 kg mol^{-1}) and one boronate group per chain, the calculated B_{short} masses required for these ratios are 228.3, 114.1, 68.5, 34.2, and 22.8 mg, respectively. Each sample was adjusted to a final volume of 0.600 mL with CD_2Cl_2, giving a P4VP concentration of 50.0 mg mL^{-1} and a nominal pyridine-site concentration of 0.476 mol L^{-1}. The corresponding nominal boronate concentrations were 0.317, 0.159, 0.0951, 0.0476, and 0.0317 mol L^{-1}, respectively. ^{1}H and ^{11}B NMR spectra were recorded at room temperature.

Synthesis of 2-(4-vinylphenyl)boronic acid catechol ester

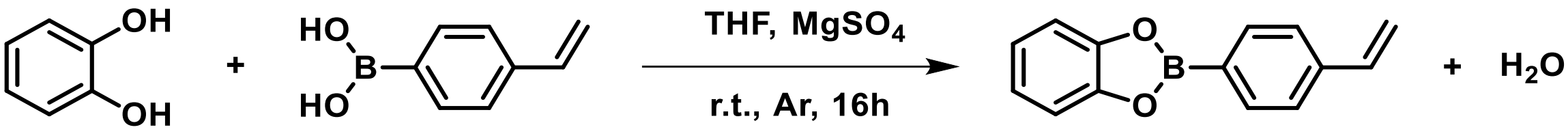

The synthesis was adapted with minor modifications from a previously reported procedure.[1] 4-Vinylphenylboronic acid (4.00 g, 26.2 mmol), catechol (2.92 g, 26.2 mmol), and magnesium sulfate (4.0 g) were placed in a 250 mL flask inside a glovebox. Dry THF (100 mL) was added, and the resulting suspension was stirred for 24 h. The suspension was then filtered through a fritted glass filter (porosity 4), and the solvent was removed from the yellowish-orange filtrate under reduced pressure. *n*-Heptane (140 mL) was added to the residue, and the mixture was sonicated for 3 h in an ultrasonic bath. The resulting suspension was filtered and subsequently centrifuged. The clear supernatant was collected, and the solvent was removed under reduced pressure to afford the product as a white solid.

Yield: 86%; ^{1}H-NMR (300 MHz, $CDCl_3$, δ): 5.38 (d, J = 10.9 Hz, 1H), 5.89 (d, J = 17.6 Hz, 1H), 6.79 (dd, J1 = 17.7 Hz, J2 = 11.0 Hz, 1H), 7.13 (dd, J1 = 9.2 Hz, J2 = 2.5 Hz, 2H), 7.3 (dd, J1 = 9.2 Hz, J2 = 2.5 Hz, 2H), 7.53 (d, J = 8.0 Hz, 2H), 8.06 (d, J = 8.1 Hz, 2H) ppm.

^{11}B-NMR (96 MHz, $CDCl_3$, δ): 32.2 ppm.

Synthesis of α-(catechol boronic ester)-terminated PDMS (B_{Long} and B_{Short})

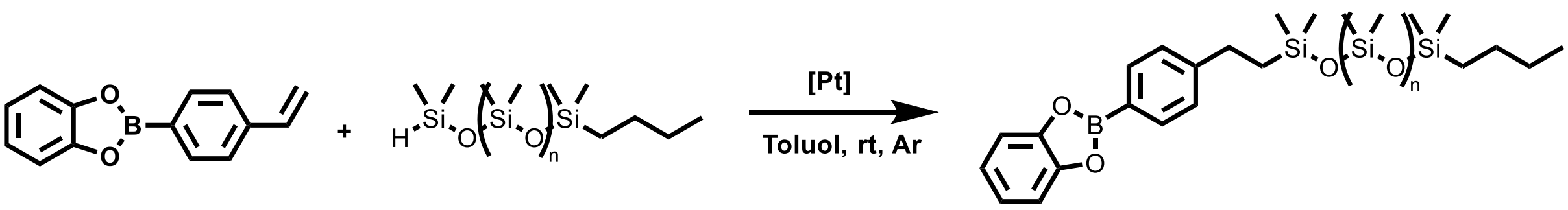

Monohydride-terminated polydimethylsiloxane (MCR-H21, 6.0 g, 0.95 mmol) and (4-vinylphenyl)boronic acid catechol ester (0.25 g, 1.12 mmol) were dissolved separately in dry toluene (7 mL total) and combined under an argon atmosphere. In a glovebox, Karstedt catalyst (2 wt% in xylene, 4 µL) was added, resulting in a pale yellow solution. The reaction mixture was stirred at room temperature for 24 h. Activated carbon (20 mg) was then added, and stirring was continued overnight at room temperature. The reaction mixture was subsequently filtered through a pad of dry Celite, and the solvent was removed under reduced pressure. The residue was washed with acetonitrile (3 × 10 mL) using vigorous shaking and sonication. After each washing step, the suspension was centrifuged, and the supernatant was discarded. The purified product was dried under reduced pressure to afford α-(catechol boronic ester)-terminated PDMS (B_{Long}) as a clear, viscous liquid. B_{short} was prepared analogously using MCR-H07 (Mn ≈ 1,000 g mol^{-1}), with reagent quantities adjusted according to the polymer molar mass. The corresponding ^{1}H NMR spectrum is shown in Figure SI 4.

Yield: 81%; ^{1}H NMR (300 MHz, $CDCl_3$): δ = 7.87–7.78 (m, 2H), 7.25–7.15 (m, 4H), 6.98 (s, 2H), 2.72 (t, 2H), 2.08 (m, 1H), 1.50–1.30 (d, 3H), 0.95 (m, 2H), 0.50–0.00 (br, 724H) ppm.

^{11}B-NMR (96 MHz, $CDCl_3$, δ): 31.6 ppm.

^{29}Si-NMR (60 MHz, $CDCl_3$, δ): 7.73–7.15, 5.44, −21.96 to −20.97 ppm.

Synthesis of α,ω-bis(catechol boronic ester)-terminated PDMS[2]

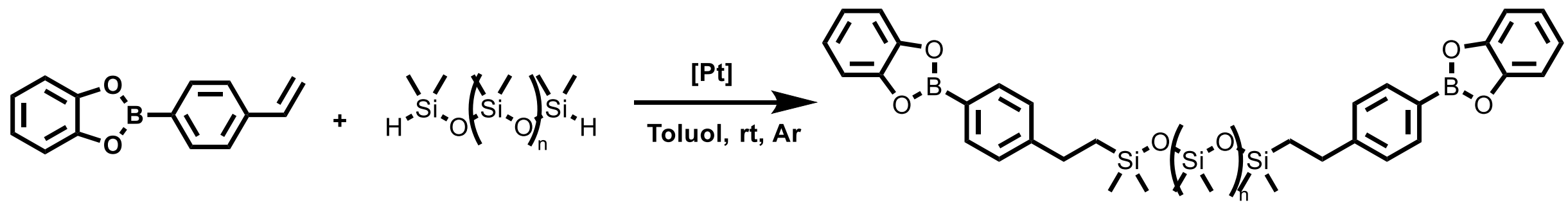

Hydride-terminated polydimethylsiloxane (DMS-H25, 6.0 g, 0.42 mmol) and (4-vinylphenyl)-boronic acid catechol ester (0.23 g, 1.04 mmol) were dissolved separately in dry toluene (7 mL total) and combined under an argon atmosphere. In a glovebox, Karstedt catalyst (2 wt% in xylene, 5 µL) was added, resulting in a pale yellow solution. The reaction mixture was stirred at room temperature for 24 h. Activated carbon (~ 0.5 g) was then added, and stirring was continued overnight at room temperature. The reaction mixture was subsequently filtered through a pad of dry Celite, and the solvent was removed under reduced pressure. The residue was washed with acetonitrile (3 × 15 mL) using vigorous shaking and sonication. After each washing step, the suspension was centrifuged, and the supernatant was discarded. The purified product was dried under reduced pressure to afford α,ω-bis(catechol boronic ester)-terminated PDMS as a clear, viscous liquid.

Yield: 79%; ^{1}H NMR (300 MHz, $CDCl_3$): δ = 7.96–7.86 (m, 4H), 7.28–7.12 (m, 8H), 6.99 (s, 4H), 2.65 (t, 4H), 2.23 (m, 2H), 1.36 (d, 6H), 0.91 (m, 4H), 0.50–0.00 (br, 1428H) ppm.

^{11}B-NMR (96 MHz, $CDCl_3$, δ): 31.9 ppm.

^{29}Si-NMR (60 MHz, $CDCl_3$, δ): 7.71–7.15, −21.96 to −20.97 ppm.

Representative Preparation of Graft-Containing Formulations and Elastomers.

Two preparation routes were employed depending on the viscosity of the formulation. P4VP and the corresponding monofunctional PDMS–boronate component were dissolved separately in dry dichloromethane (DCM), combined in the desired ratio, and stirred at room temperature for 30–60 min until homogeneous. After slow evaporation of DCM at room temperature for 24–48 h, sufficiently fluid formulations were converted into elastomers by direct addition and homogeneous incorporation of the difunctional boronic ester crosslinker without further solvent. For more viscous formulations, solvent-free mixing was impractical; the crosslinker was therefore added to the PDMS–boronate solution before combination with P4VP, and the complete formulation was cast from DCM. All samples were subsequently dried under vacuum at room temperature for 1–2 h and at 50 °C for at least 12 h, washed with DMF, and dried again under reduced pressure prior to characterization. The final elastomer formulations were transferred into PTFE molds with internal dimensions of 10 × 40 × 2 mm for tensile specimens or into cylindrical PTFE molds with an internal diameter of 10 mm and a depth of 2 mm for rheological specimens.

The NMR-derived side-chain loading was calculated from an isolated two-proton boronate-aryl resonance and a two-proton P4VP aromatic resonance: $\theta_{NMR} = (I_{boronate}/I_{P4VP}) \times 100\%$. Thus, θ_{NMR} reports the molar ratio of PDMS–boronate chains to 4VP repeat units and not the instantaneous fraction of B–N-coordinated sites.

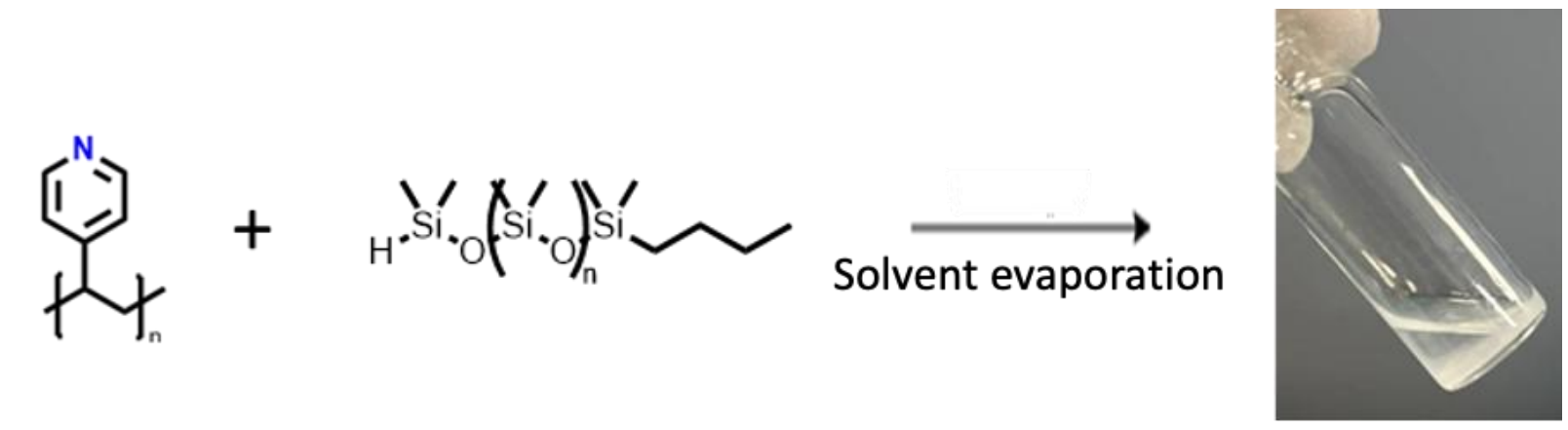

Figure SI 1: Control experiment with unfunctionalized PDMS and P4VP. The schematic representation illustrates the absence of complementary Lewis acid–base functionalities. Upon mixing under the same conditions used for the boronate-functionalized PDMS/P4VP formulations, macroscopic phase separation was observed, with precipitation of P4VP and formation of a PDMS-rich liquid phase. This result supports that boronate–pyridine coordination is required for compatibilization and formation of homogeneous dynamically grafted polymer formulations under the employed conditions.

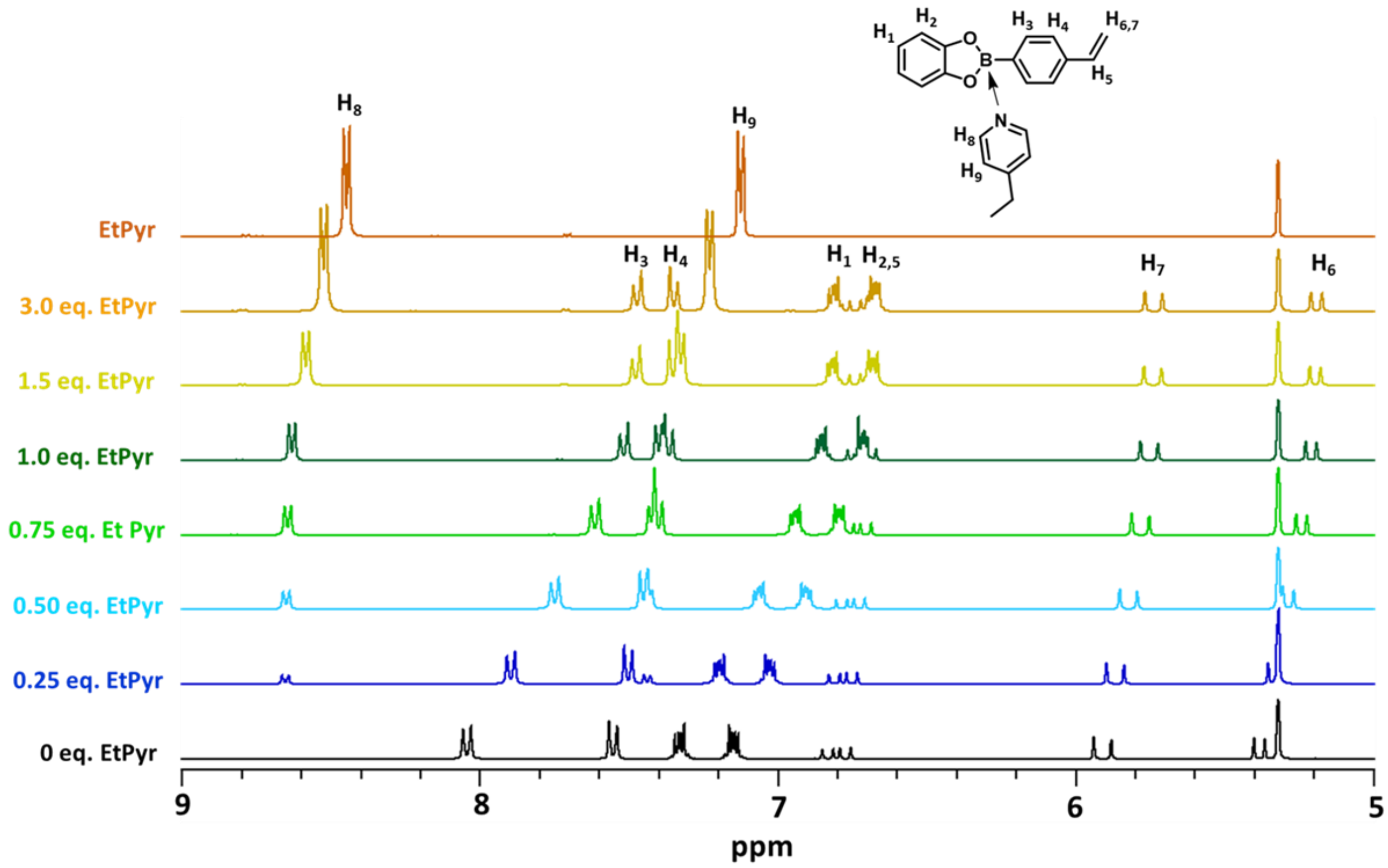

Exp.	[EtPyr] / Eq.	H3 δ / ppm	H3 Δδ / ppm
1	0.00	8.0435	0.000
2	0.25	7.8960	0.148
3	0.50	7.7480	0.296
4	0.75	7.6130	0.430
5	1.00	7.5165	0.527
6	1.50	7.4760	0.568
7	3.00	7.4710	0.573

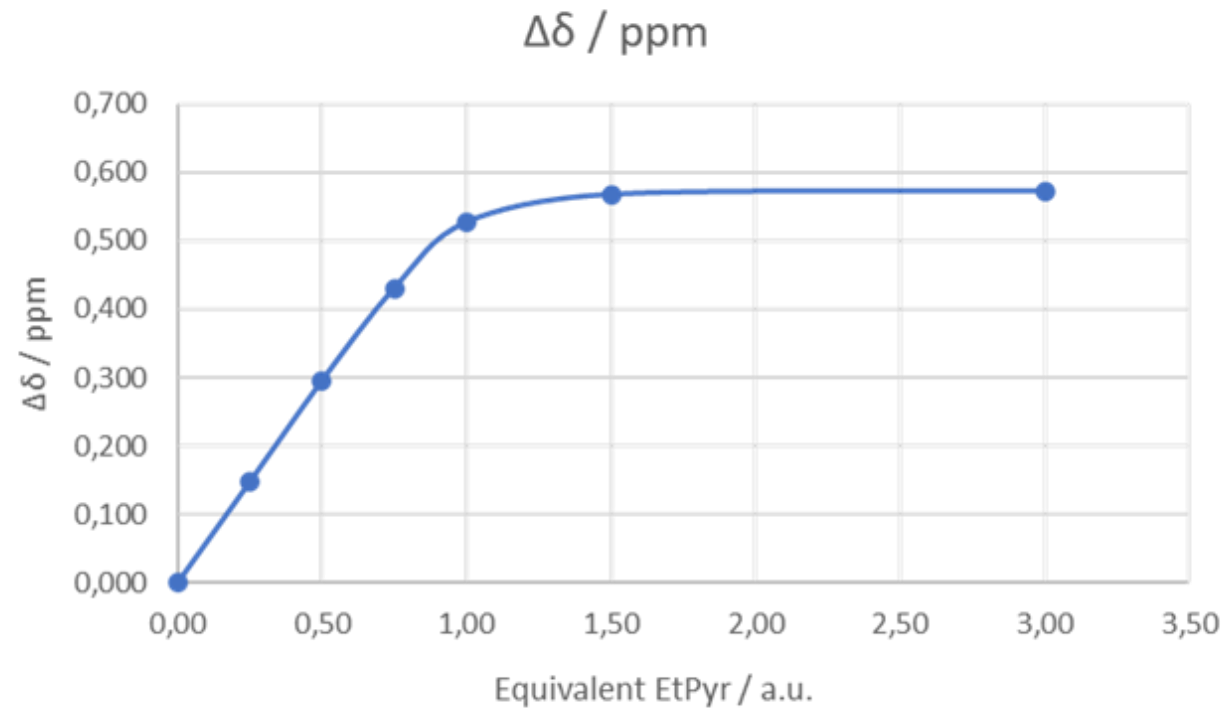

Figure SI 2: ^1H NMR spectra of 2-(4-vinylphenyl)boronic acid catechol ester upon addition of increasing amounts of 4-ethylpyridine in CD_2Cl_2 at room temperature. Lewis base equivalents are given relative to the boronic ester. The composition-dependent shifts of the boronate-adjacent aromatic resonances H_3 and H_4 are consistent with progressive B–N coordination. The inset shows the chemical-shift change of H_3 as a function of Lewis base equivalents.

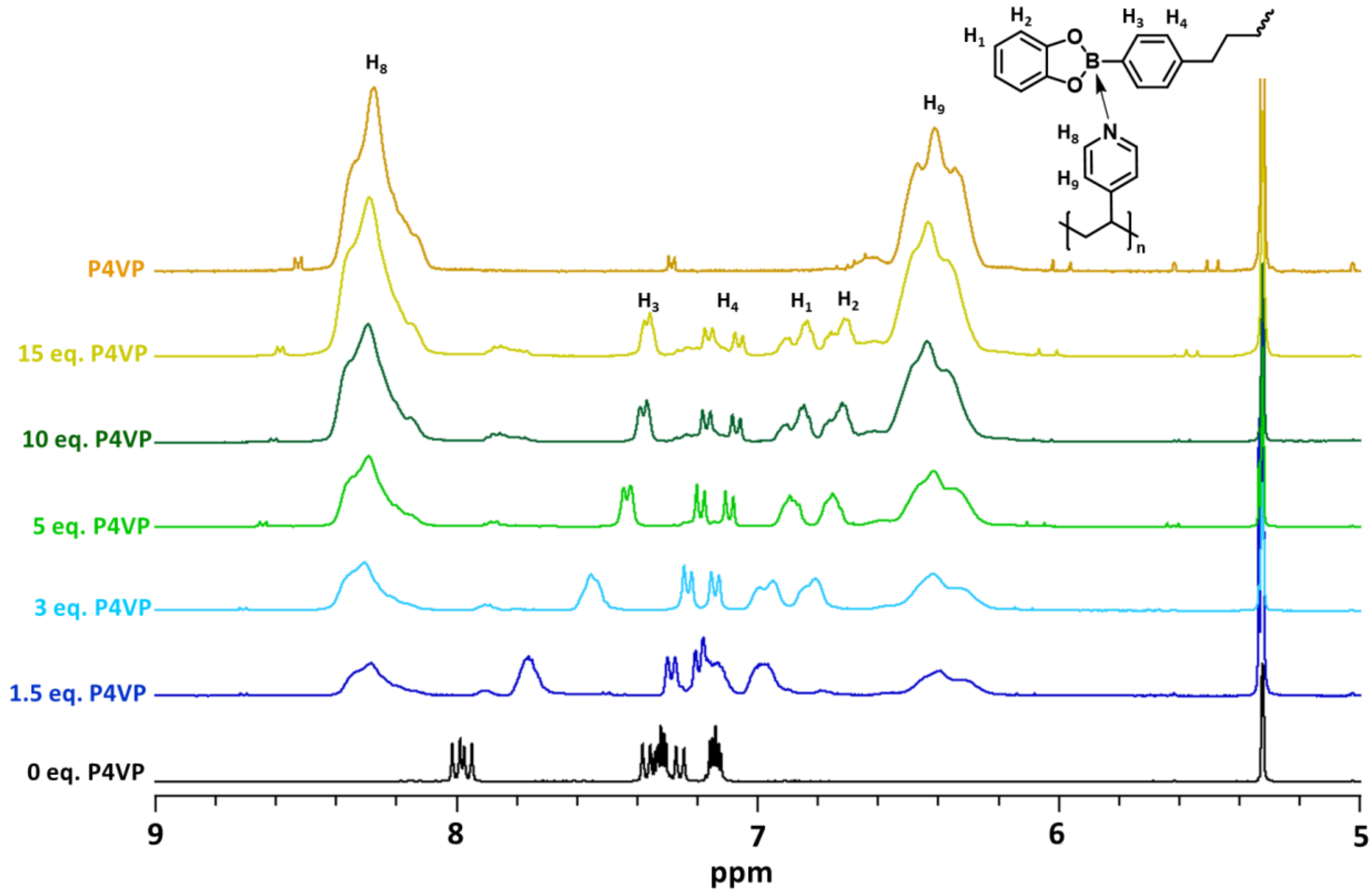

Figure SI 3: ^{1}H NMR spectra of the B_{short} polymer system prepared by mixing catechol boronate ester-functionalized PDMS with increasing amounts of P4VP in CD_2Cl_2 at room temperature. P4VP equivalents refer to pyridine repeat units relative to boronic ester groups. The spectra of the individual P4VP and boronate-functionalized PDMS components are shown at the top and bottom, respectively. The composition-dependent shifts and broadening of the boronate-adjacent aromatic resonances H_3 and H_4 are consistent with progressive B–N coordination within the heterogeneous macromolecular environment.

Table SI 1: **Nominal and NMR-derived compositions of the dynamically grafted polymer series.** L/S denotes the molar ratio of long to short PDMS side chains. The side-chain number-average molar masses, $M_{n,SC}$, were estimated from the supplier-reported molar masses of the monohydride-terminated PDMS precursors by adding 222 g mol^{-1} for the boronate end group, assuming complete end functionalization. The nominal side-chain loading, θ_{nom}, is defined as the molar ratio of monofunctional PDMS–boronate chains to 4VP repeat units used in the formulation, expressed as a percentage. The corresponding composition ratio determined from ^{1}H NMR integrations is denoted θ_{NMR}. Both quantities describe side-chain-to-4VP composition ratios and do not directly quantify the fraction of coordinated side chains. The deviation, $\Delta\theta=\theta_{NMR}-\theta_{nom}$,

is expressed in percentage points. The P4VP content (wt%) represents the mass percentage of P4VP in the total solvent-free formulation, calculated from the amounts of P4VP and PDMS–boronate side chains used.

Polymer	L/S molar ratio	$M_{n,SC}$ / kDa	θ_{nom} / %	θ_{NMR} / %	Δθ / percentage points	P4VP content / wt%
**BL-10**	100 / 0	6.5	10	9	-1	13.9
**BL-30**	100 / 0	6.5	30	30	0	5.1
**BL-50**	100 / 0	6.5	50	47	-3	3.1
**BL-70**	100 / 0	6.5	70	67	-3	2.3
**BL-90**	100 / 0	6.5	90	-	-	1.8
**BS-10**	0 / 100	1.2	10	10	0	46.2
**BS-30**	0 / 100	1.2	30	30	0	22.3
**BS-50**	0 / 100	1.2	50	48	-2	14.7
**BS-70**	0 / 100	1.2	70	71	1	10.9
**BS-90**	0 / 100	1.2	90	-	-	8.7
**BM-L90S10**	90 / 10	6.5 / 1.2	50	-	-	3.4
**BM-L70S30**	70 / 30	6.5 / 1.2	50	-	-	4.1
**BM-L50S50**	50 / 50	6.5 / 1.2	50	-	-	5.2

Table SI 2: Molecular characteristics of the polymeric components used in this study. Molar masses of the commercial PDMS precursors are supplier-reported number-average molar masses M_n, whereas the P4VP value corresponds to the supplier-reported weight-average molar mass M_w. The molar masses of the boronate-functionalized PDMS derivatives were estimated from the corresponding precursor molar masses by accounting for the introduced boronate end group(s), assuming quantitative end functionalization. N_{SC} denotes the approximate number of dimethylsiloxane repeat units per PDMS chain, while N_{BB} denotes the weight-average number of 4VP repeat units in the P4VP backbone estimated from the supplier-reported M_W.

Component	Supplier/material	Molar mass / kDa	N_{SC} or N_{BB}	Functionality
P4VP	Sigma 472352	160 (Mw)	≈1500	pyridine
MCR-H07	Gelest	1 (Mn)	≈14	1 Si–H
B_{short}	synthesized	1.2 (estimated M_n)	≈14	1 B
MCR-H21	Gelest	6.3 (Mn)	≈85	1 Si–H
B_{long}	synthesized	6.5 (estimated M_n)	≈85	1 B
DMS-H25	Gelest	14.4 (Mn)	≈195	2 Si–H
Difunctional linker	synthesized	14.6 (estimated M_n)	≈195	2 B

Calculation of the PDMS-matched control formulation C

Formulation C was designed to match the total mass of PDMS-based components in formulation B while containing no monofunctional PDMS–boronate side chains. The calculation was performed on the basis of 100 mol of 4VP repeat units.

Formulation B contains 1.00 mol of B_{long}, corresponding to θ_{nom} = 1%, and 1.50 mol of the difunctional linker, because a crosslinker-derived B loading of 3.0% corresponds to 3.00 mol of boronate groups and each linker molecule contributes two boronate groups.

Using M_n = 6.5 kg mol^{-1} for B_{long} and M_n = 14.6 kg mol^{-1} for the difunctional linker, the total mass of PDMS-based components in formulation B is:

$m_{PDMS,B}$ = (1.00 × 6.5) + (1.50 × 14.6) = 28.4 kg.

To introduce the same mass using only the difunctional linker, formulation C requires:

$n_{linker,C}$ = 28.4 / 14.6 = 1.945 mol

of linker per 100 mol of 4VP repeat units. Because each linker molecule contains two boronate groups, this corresponds to a crosslinker-derived B loading of:

(2 × 1.945 / 100) × 100% = 3.89% ≈ 3.9%.

Accordingly, the crosslinker-derived B loading of formulation C was set to 3.9% (Table 1).

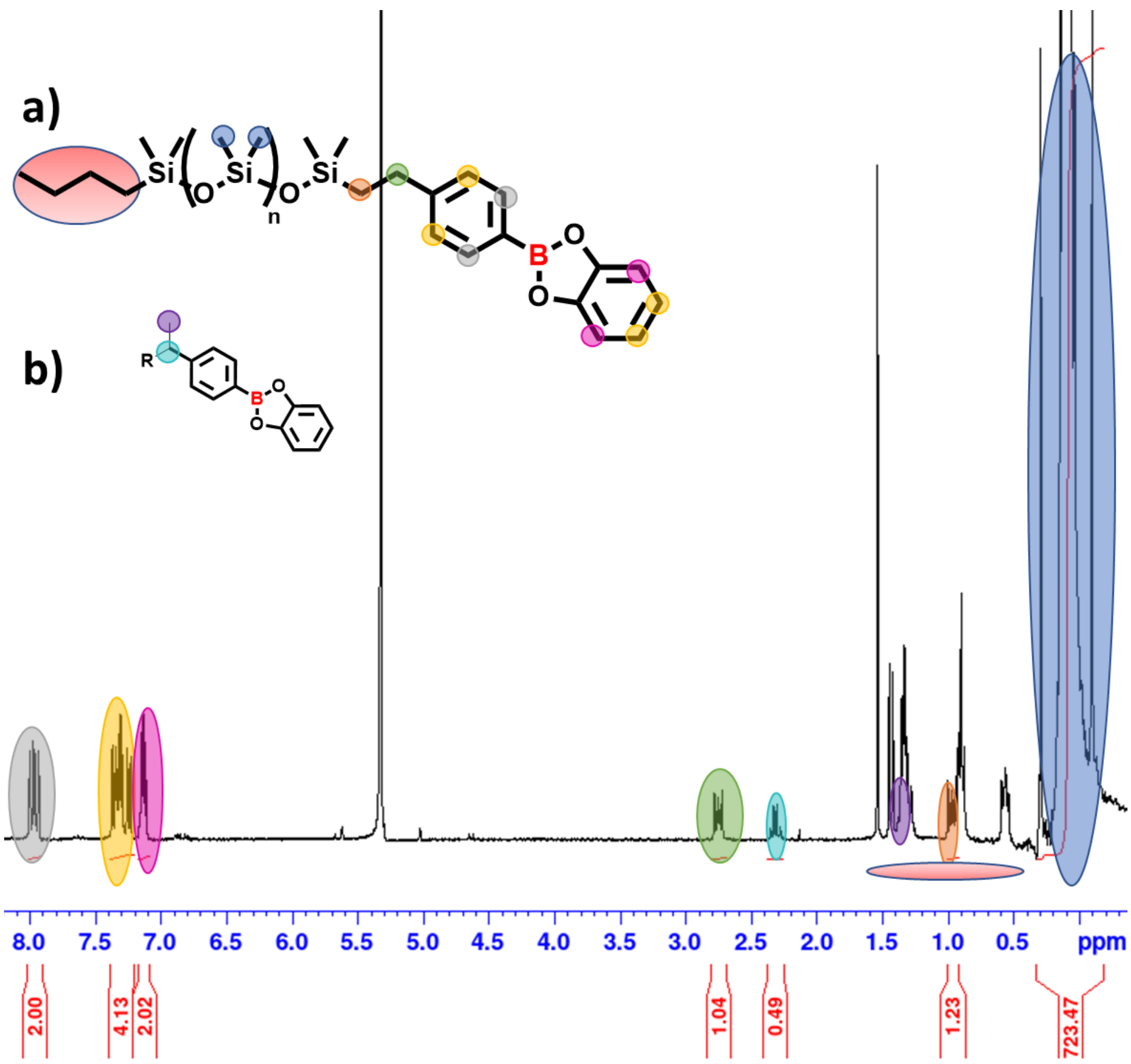

Figure SI 4: ^{1}H NMR spectrum of α-(catechol boronic ester)-terminated PDMS (Bshort) recorded in $CDCl_3$. Selected resonance assignments are highlighted.

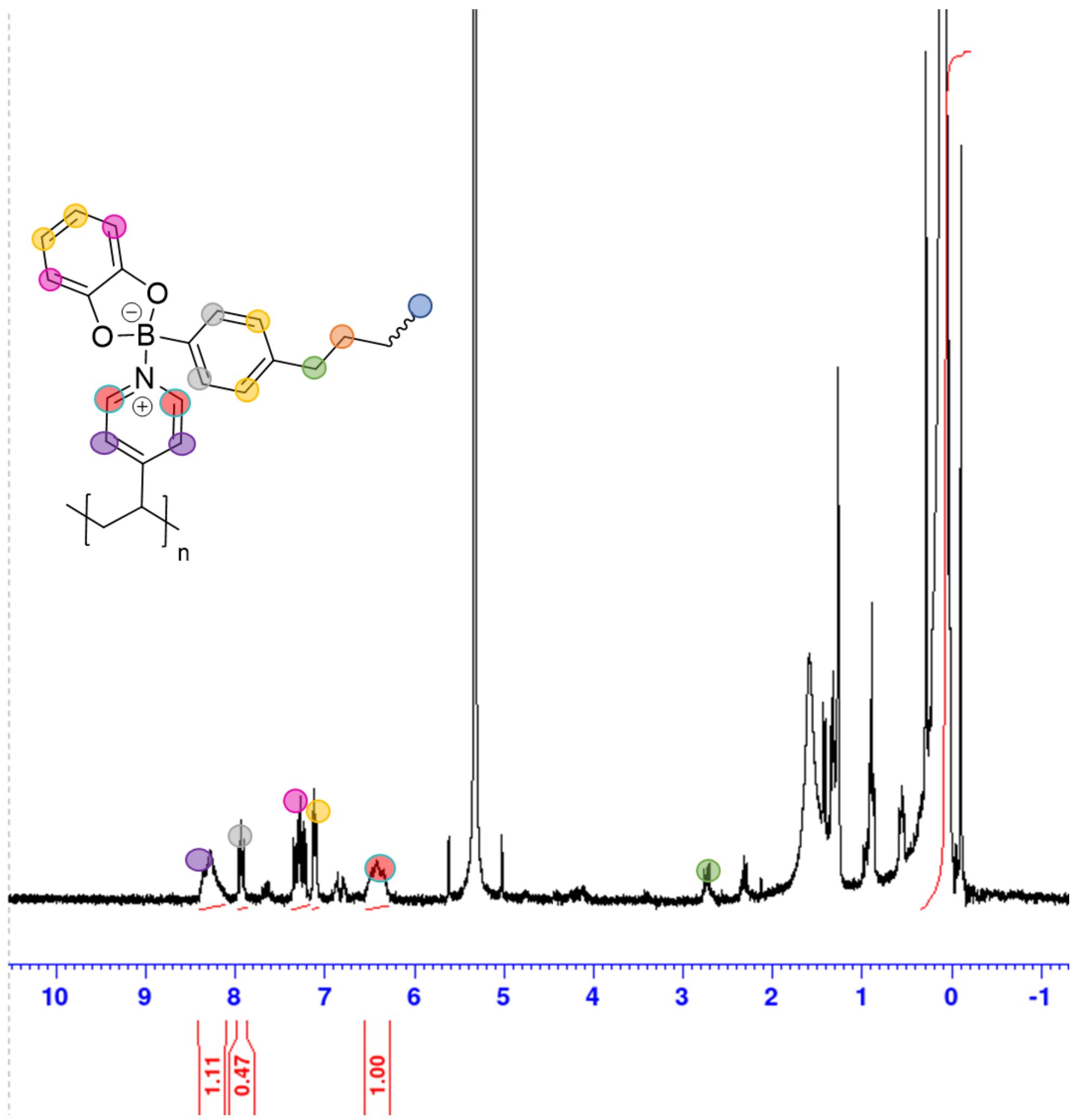

Figure SI 5: Representative ^{1}H NMR spectrum of a B_{long} sample (BL-50) used to determine the NMR-derived side-chain loading θ_{NMR}. The integral of a two-proton boronate-aryl resonance was referenced to that of a two-proton P4VP aromatic resonance to determine the molar ratio of PDMS–boronate side chains to 4VP repeat units, expressed as a percentage. The corresponding compositions are summarized in Table SI 1.

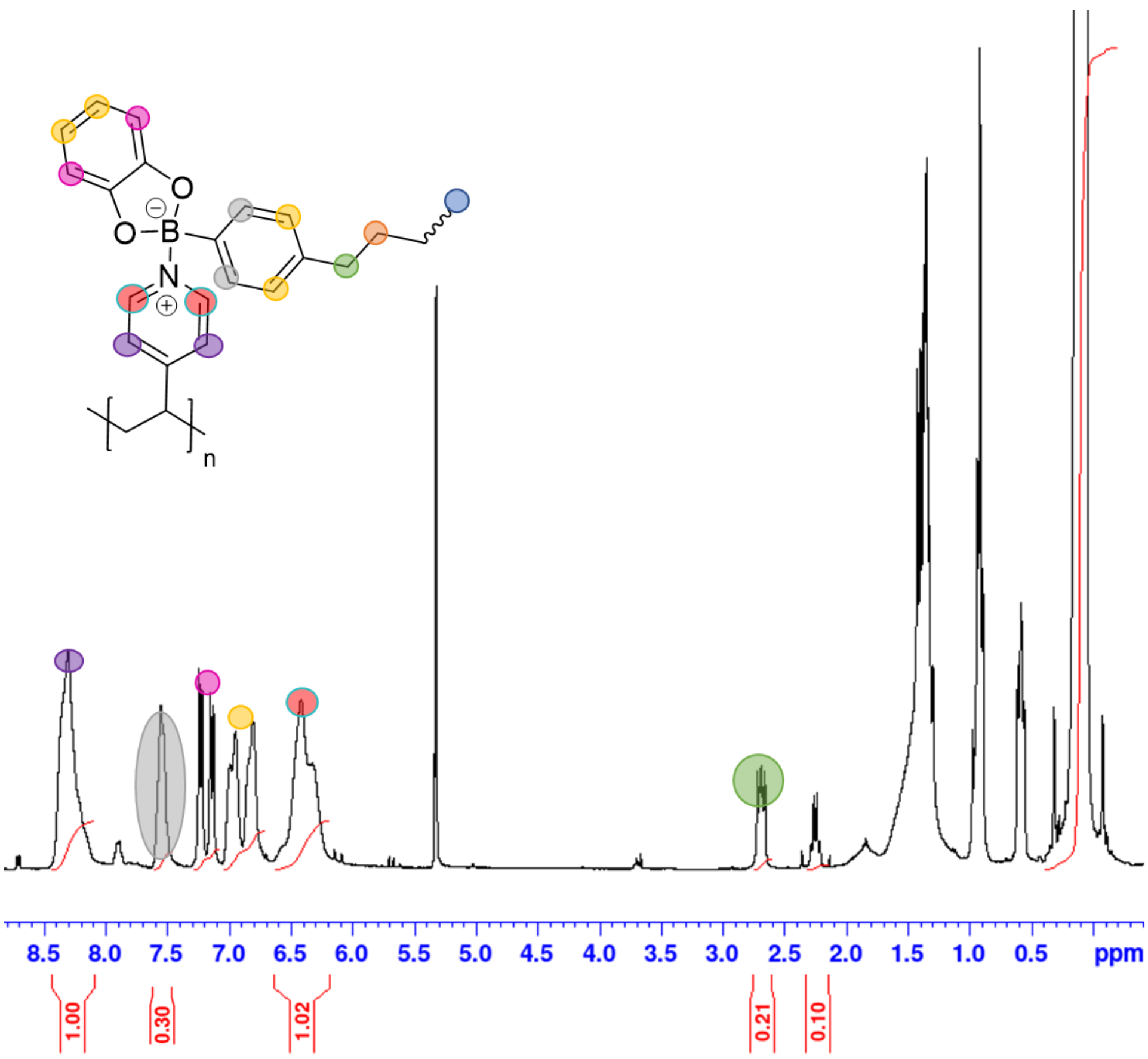

Figure SI 6: Representative ^{1}H NMR spectrum of a B_{short} sample (BS-30) used to determine the NMR-derived side-chain loading θ_{NMR}. The integral of a two-proton boronate-aryl resonance was referenced to that of a two-proton P4VP aromatic resonance to determine the molar ratio of PDMS–boronate side chains to 4VP repeat units, expressed as a percentage. The corresponding compositions are summarized in Table SI 1.

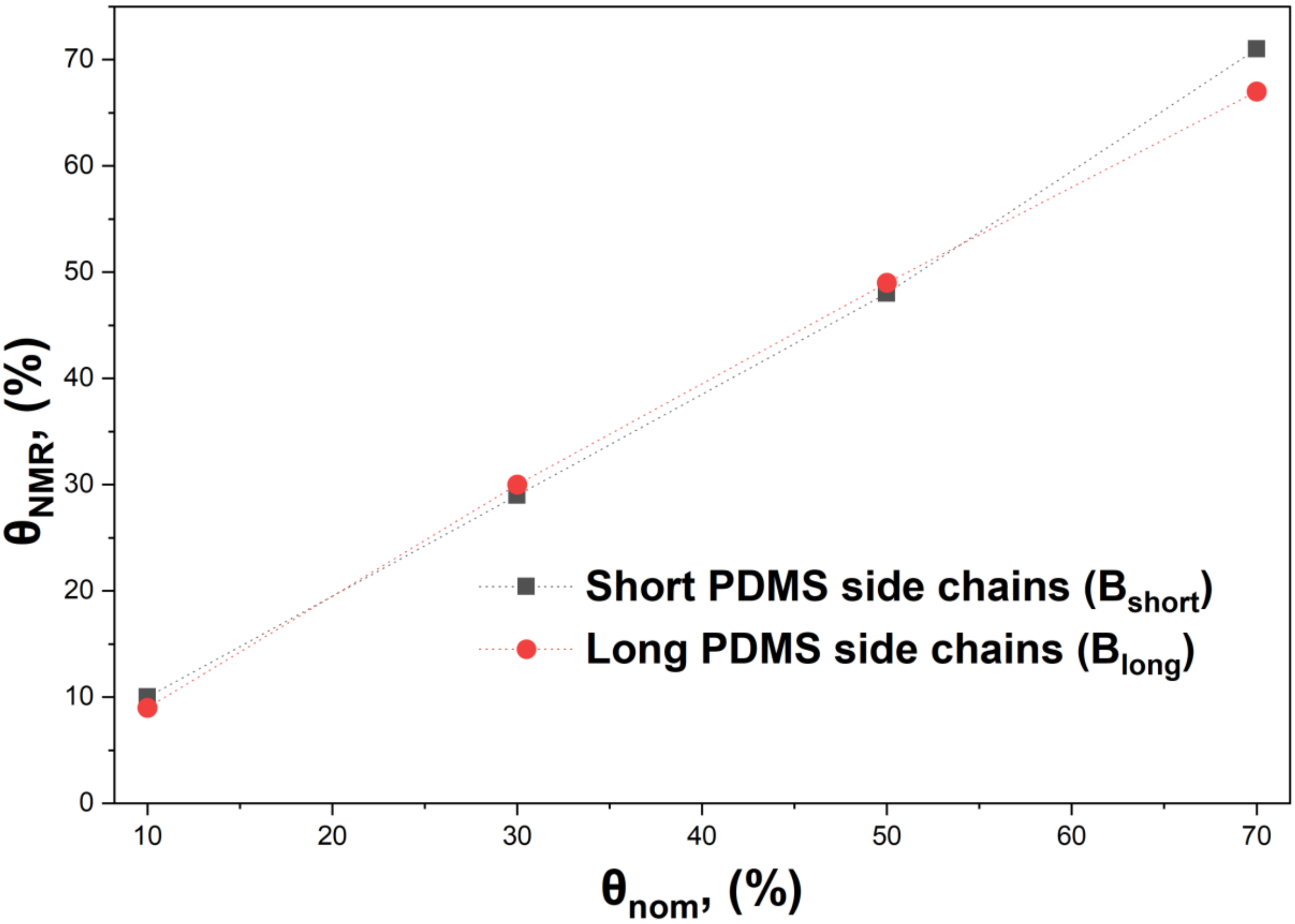

Figure SI 7: Comparison of the nominal side-chain loading, θ_{nom}, with the composition ratio determined by ^{1}H NMR spectroscopy, θ_{NMR}, for the B_{short} and B_{long} polymer series. The dotted lines connect the data points as guides to the eye. The NMR-derived composition ratios closely matched the nominal side-chain loadings, with deviations of no more than 3 percentage points over the investigated range θ_{nom} = 10–70%. Neither quantity directly measures the fraction of coordinated side chains.

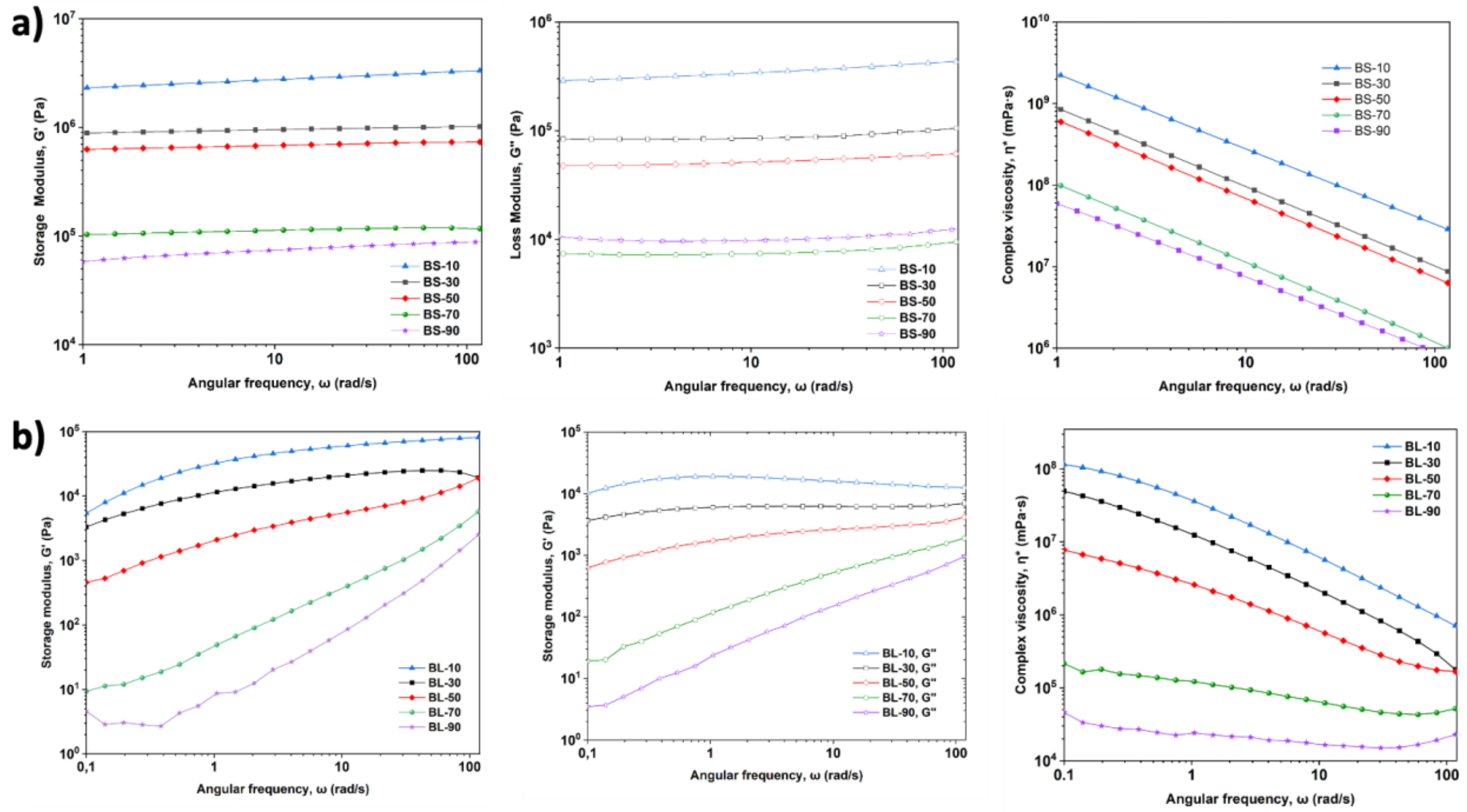

Figure SI 8: Additional oscillatory frequency sweeps for the B_{short} and B_{long} polymer series. The data include the intermediate nominal loading (θnom = 30%), which is omitted from Figure 2 for clarity, together with the θnom = 90% samples. Storage modulus (G′), loss modulus (G″), and complex viscosity (η*) are shown as a function of angular frequency.

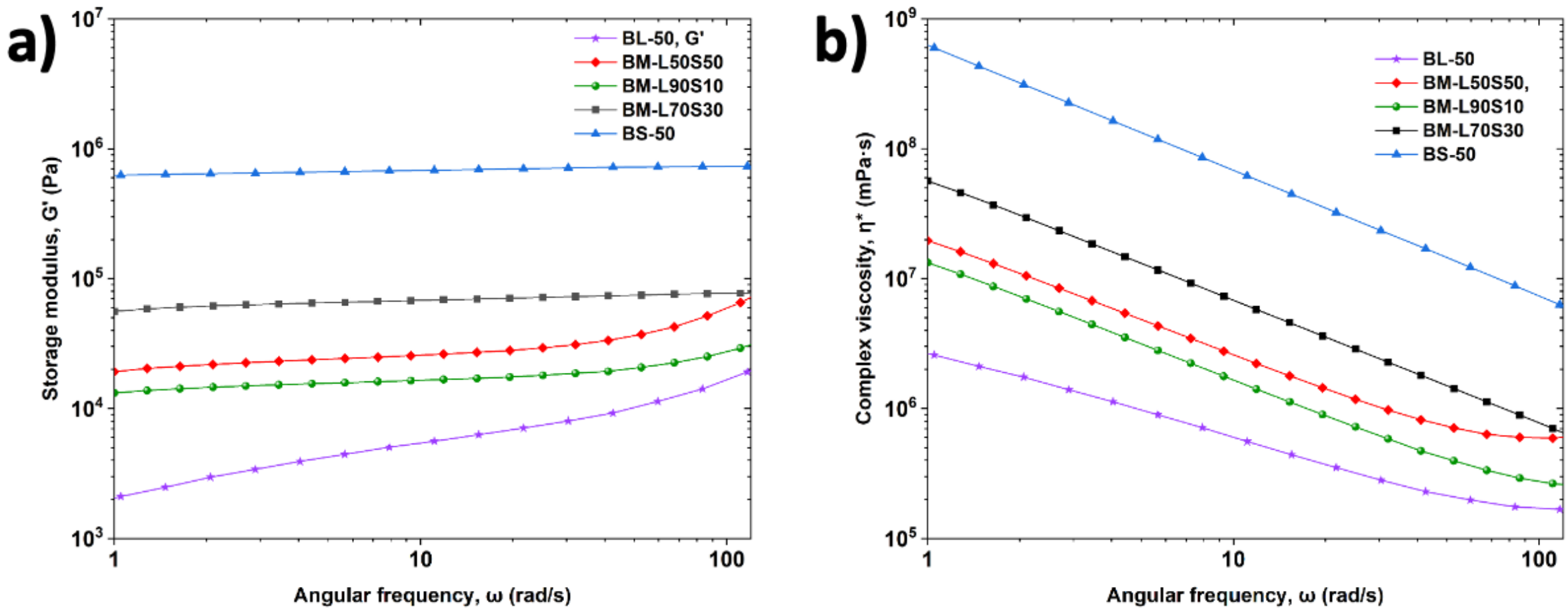

Figure SI 9: Oscillatory shear rheology of polymers with mixed PDMS side-chain compositions at a nominal side-chain loading of θnom = 50% and varying long-to-short PDMS side-chain ratios (Table SI 1). (a) Storage modulus (G′) and (b) complex viscosity (η*) are shown as a function of angular frequency. The mixed side-chain formulations exhibit intermediate viscoelastic properties between the corresponding B_{short} and B_{long} analogues.

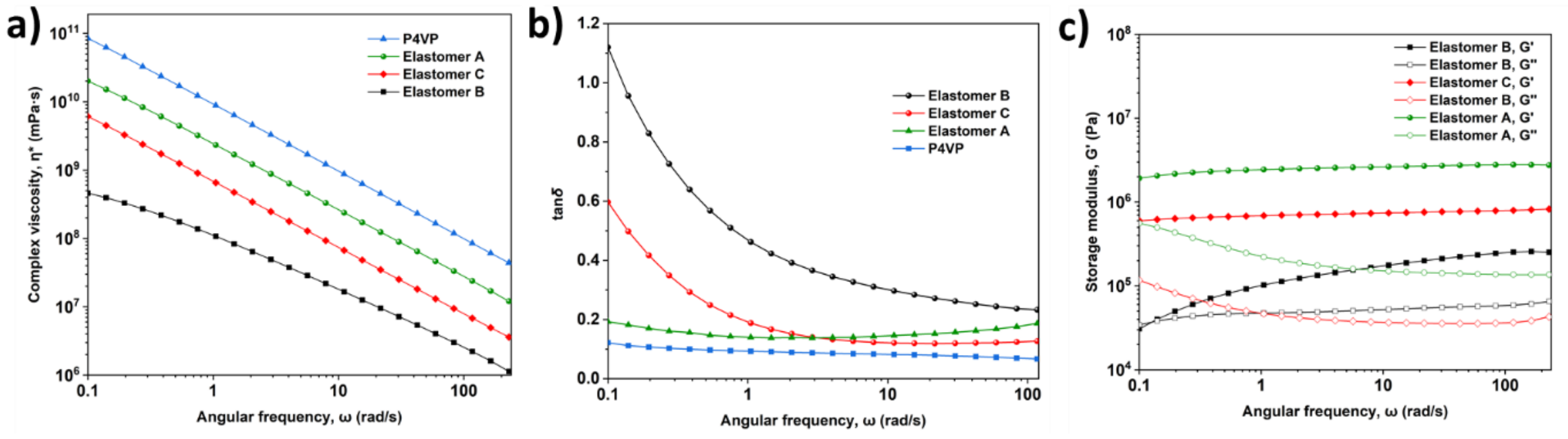

Figure SI 10: Additional characterization of the dynamically crosslinked elastomer systems. (a) Complex viscosity (η*), (b) loss factor (tan δ), and (c) storage modulus (G′, filled symbols) and loss modulus (G″, open symbols) as a function of angular frequency for elastomer formulations with different crosslink densities.

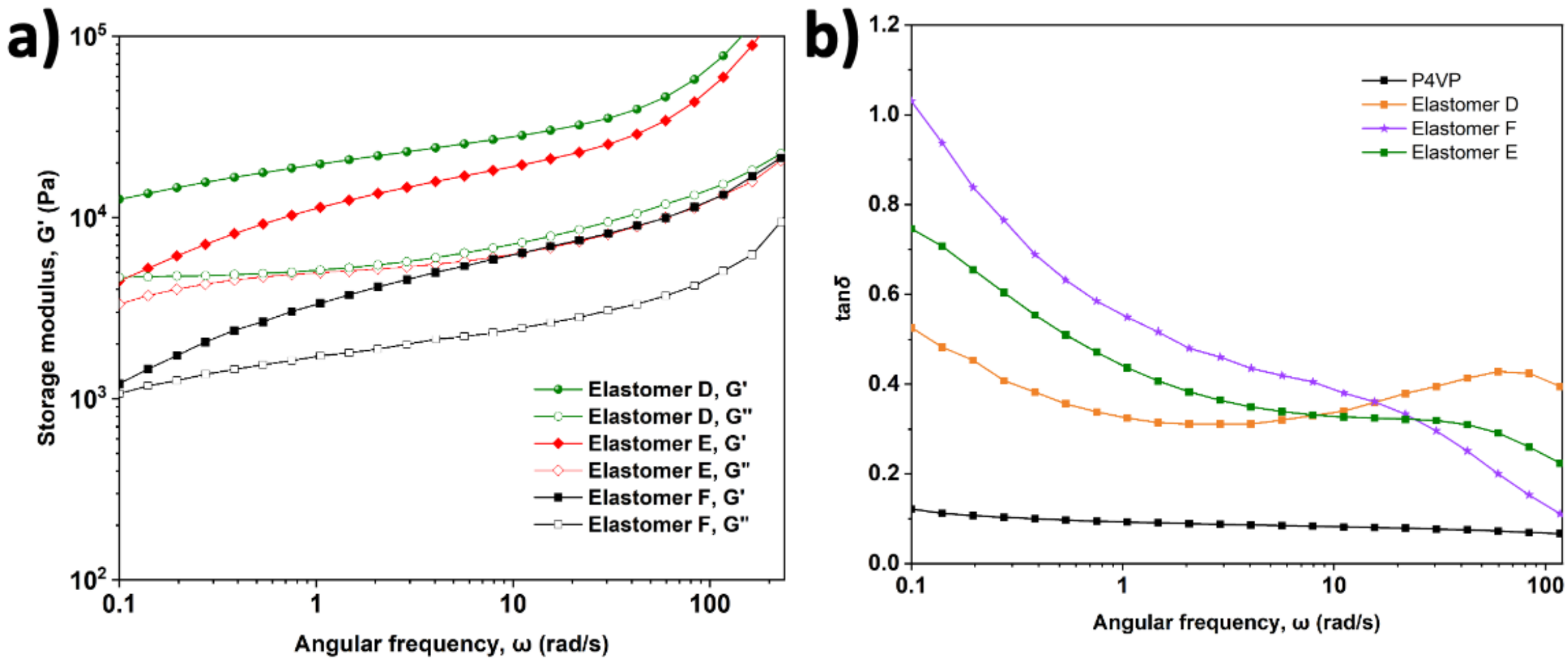

Figure SI 11: Oscillatory shear rheology of the second elastomer series with reduced P4VP content. (a) Storage modulus (G′, filled symbols) and loss modulus (G″, open symbols) and (b) loss factor (tan δ) as a function of angular frequency for Elastomers D–F. P4VP is included as a reference.

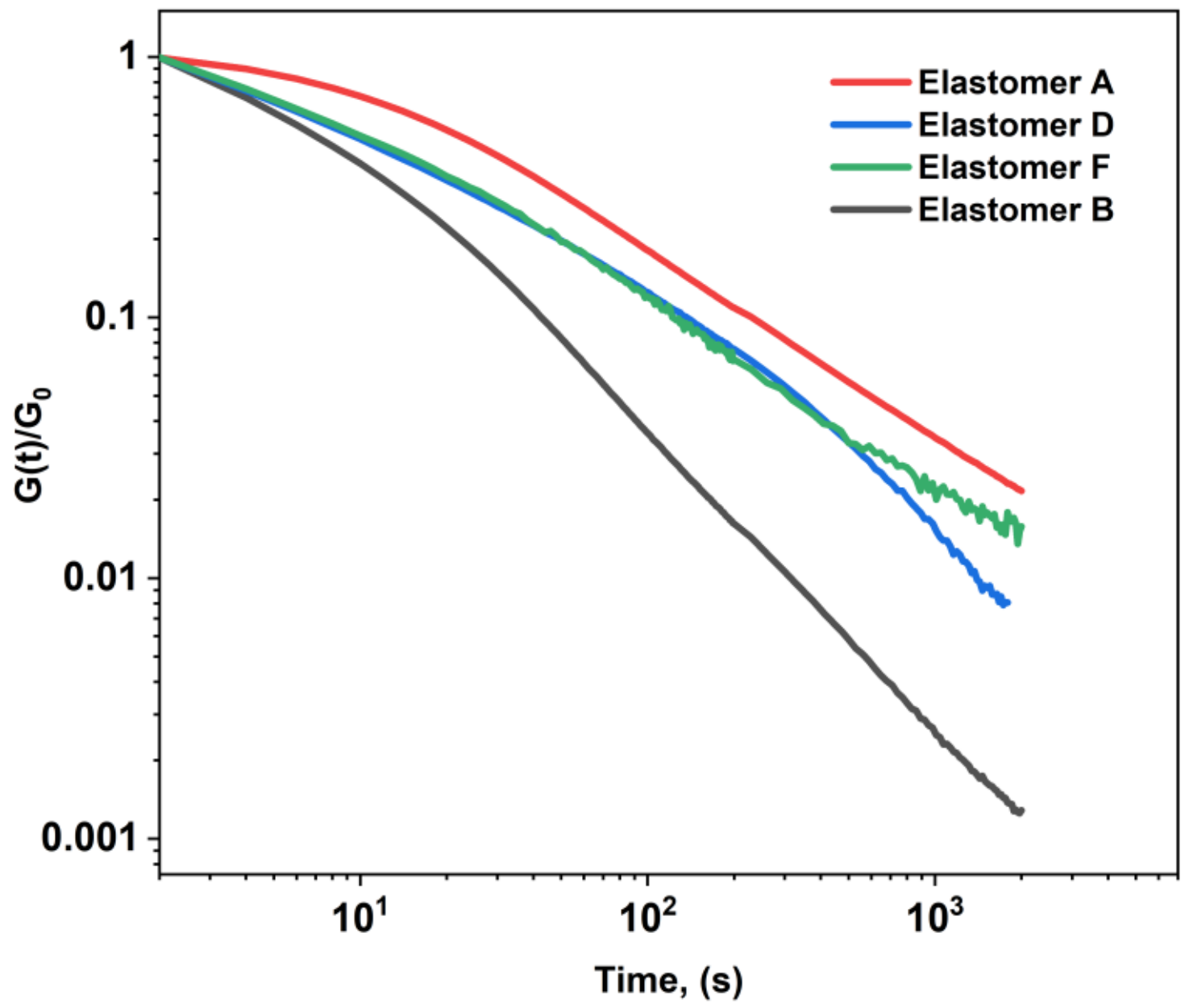

Figure SI 12: Stress relaxation of selected elastomer formulations. Normalized relaxation modulus, $G(t)/G_0$, as a function of time for Elastomers A, B, D, and F measured under a constant applied strain in the linear viscoelastic regime.

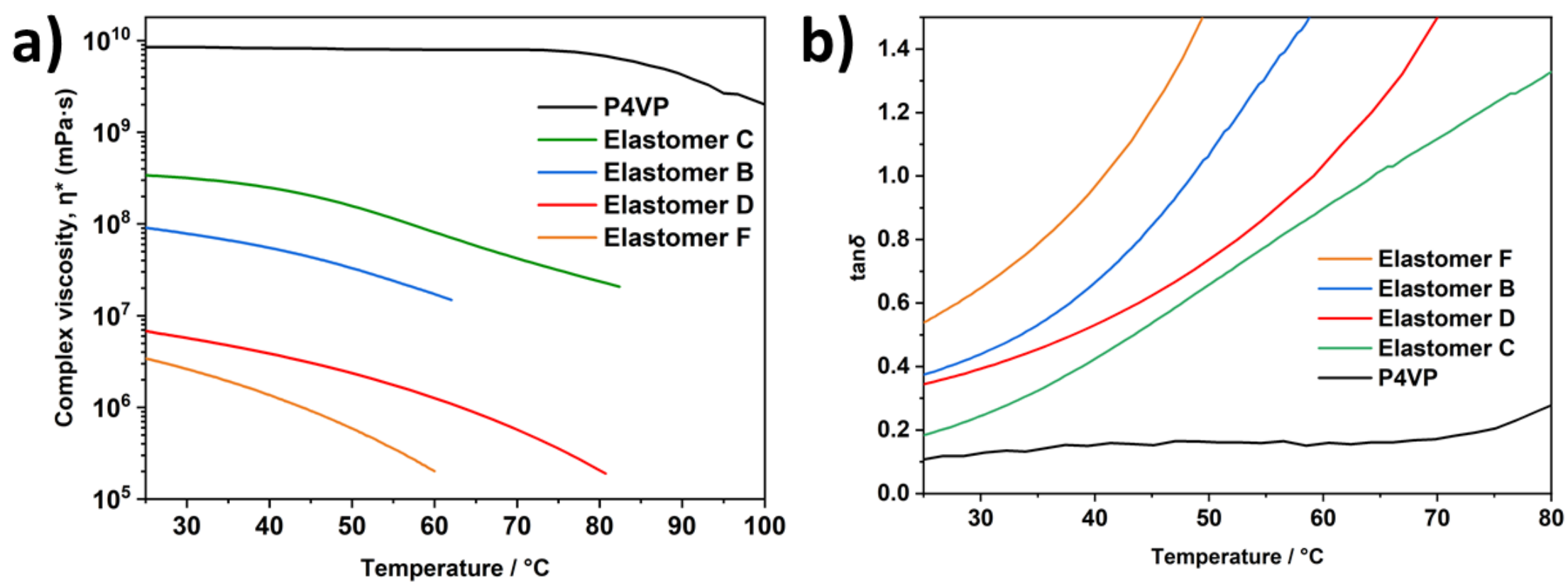

Figure SI 13: Additional characterization of the dynamically crosslinked elastomer formulations. (a) Complex viscosity (η^*) as a function of temperature for P4VP and selected elastomer formulations. (b) Loss factor (tan δ) as a function of temperature for P4VP and selected elastomer formulations.

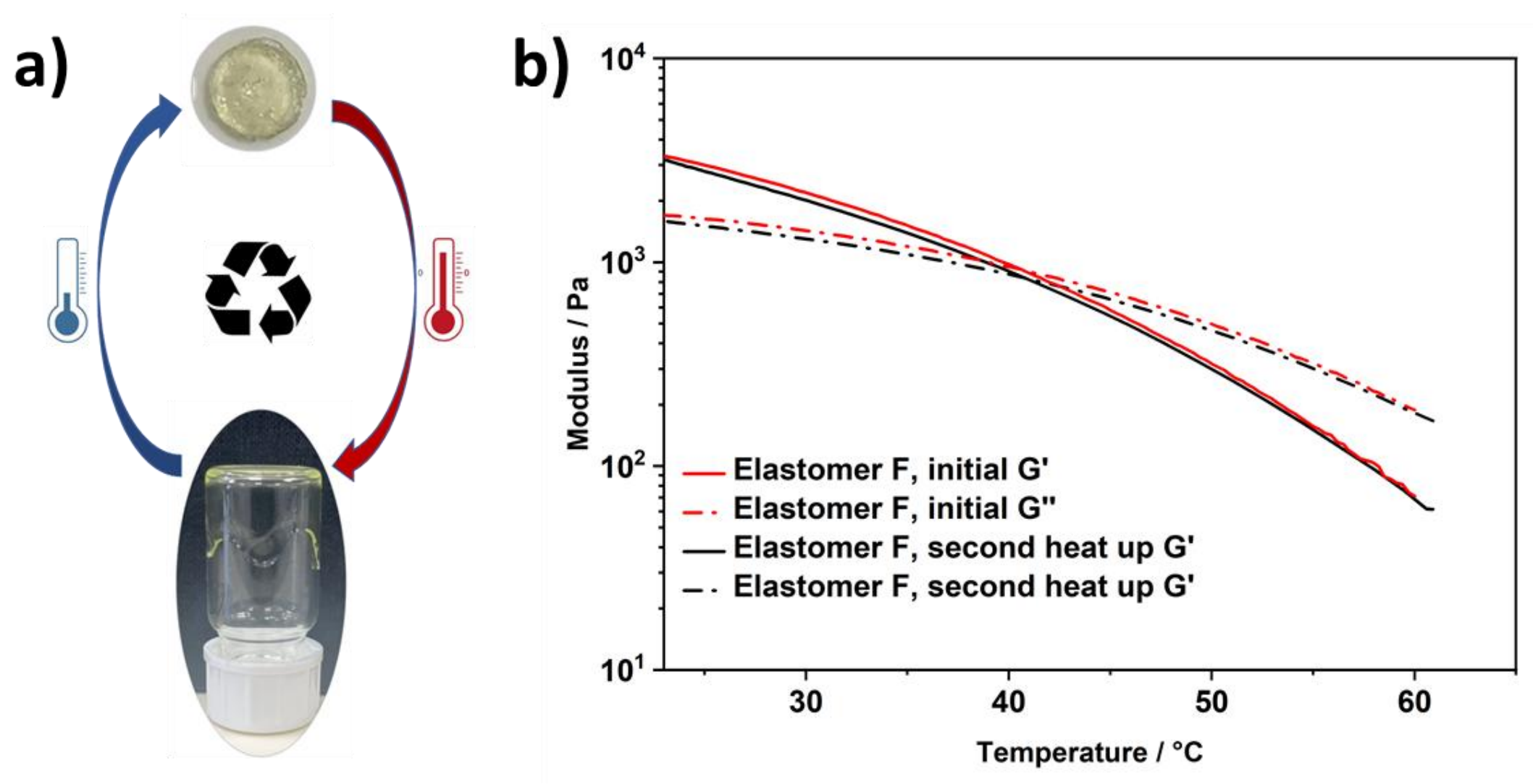

Figure SI 14: Thermal reversibility of Elastomer F. (a) Schematic illustration and photographs showing heat-induced softening and flow of the elastomer and recovery of a self-supporting material upon cooling. (b) Temperature-dependent storage modulus (G′, solid lines) and loss modulus (G″, dash-dotted lines) recorded during the initial heating cycle (red) and a second heating cycle after cooling (black), demonstrating recovery of the viscoelastic response after thermal cycling.

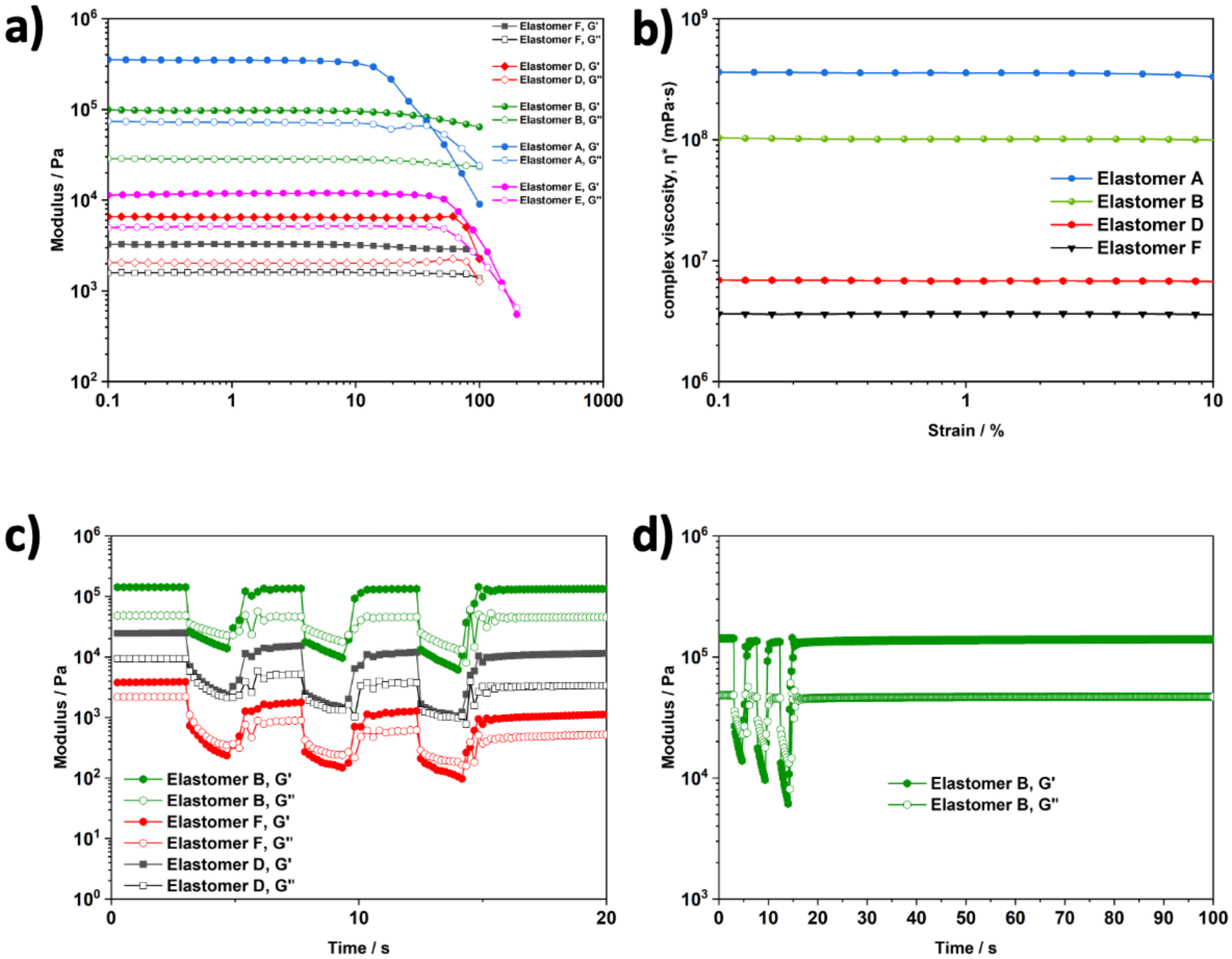

Figure SI 15: Additional rheological characterization of the dynamically crosslinked elastomer formulations. (a) Amplitude sweeps showing the storage modulus (G′, filled symbols) and loss modulus (G″, open symbols) as a function of strain amplitude for Elastomers A, B, D, E, and F. (b) Complex viscosity (η*) as a function of strain amplitude for selected elastomer formulations. (c) Step-strain measurements showing the recovery of the storage modulus (G′, filled symbols) and loss modulus (G″, open symbols) following repeated alternating low- and high-strain deformation for Elastomers B, D, and F. (d) Extended

step-strain measurement of Elastomer B demonstrating repeated recovery of G′ (filled symbols) and G″ (open symbols) after successive deformation cycles.

Estimation of the exchange timescale from the ^{11}B NMR spectra[3–5]

The observation of a single population-averaged ^{11}B resonance throughout the small-molecule titration is consistent with exchange between the uncoordinated and pyridine-coordinated boron environments being fast on the NMR timescale. An approximate lower bound for the exchange-rate was estimated from the chemical-shift separation between the limiting resonances.

The uncoordinated boronic ester showed a ^{11}B resonance at $\delta_{\mathrm{free}} = 32.2$ ppm, whereas the resonance approached $\delta_{\mathrm{coordinated}} = 11.9$ ppm at high 4-ethylpyridine loading. The resulting chemical-shift separation was

$$\Delta\delta = |\ \delta_{\mathrm{free}} - \delta_{\mathrm{coordinated}}\ | = |\ 32.2 - 11.9\ | = 20.3\ \text{ppm}.$$

At a ^{11}B observation frequency of 96 MHz, this corresponds to a frequency separation of

$$\Delta f = \Delta\delta \times f_0 = 20.3 \times 96 = 1948.8\ \text{Hz}.$$

For an ideal two-site exchange between equally populated, uncoupled resonances, the rate constant at coalescence is approximated by

$$k_{\mathrm{c}} = \frac{\pi \Delta f}{\sqrt{2}}.$$

Accordingly,

$$k_{\mathrm{c}} = \frac{\pi \times 1948.8}{\sqrt{2}} = 4.33 \times 10^{3}\ \mathrm{s}^{-1}.$$

The absence of separately resolved resonances is therefore consistent with an apparent exchange rate on the order of or greater than $4 \times 10^{3}\ \mathrm{s}^{-1}$, corresponding to a characteristic exchange timescale of approximately 0.23 ms or shorter. This value represents an order-of-magnitude estimate rather than a directly determined kinetic rate constant. The estimate assumes an idealized two-site exchange with comparable populations and does not account explicitly for quadrupolar broadening of the 11B resonances, unequal populations, nor the concentration dependence of the observed exchange rate arising from reversible association. Importantly, this local solution-state NMR timescale should not be directly equated with the terminal rheological relaxation or G'/G" crossover of the solvent-free materials, which additionally depends on chain dynamics, multichain connectivity, and composition.

References

(1) Chen, Y.; Tang, Z.; Zhang, X.; Liu, Y.; Wu, S.; Guo, B. Covalently Cross-Linked Elastomers with Self-Healing and Malleable Abilities Enabled by Boronic Ester Bonds. *ACS applied materials & interfaces* **2018**, *10* (28), 24224–24231. DOI: 10.1021/acsami.8b09863. Published Online: Jul. 5, 2018.

(2) Dodge, L.; Chen, Y.; Brook, M. A. Silicone boronates reversibly crosslink using Lewis acid-Lewis base amine complexes. *Chemistry (Weinheim an der Bergstrasse, Germany)* **2014**, *20* (30), 9349–9356. DOI: 10.1002/chem.201402877. Published Online: Jul. 1, 2014.

(3) Ishihara, K.; Nagasawa, A.; Umemoto, K.; Ito, H.; Saito, K. Kinetic Study of Boric Acid–Borate Interchange in Aqueous Solution by ^{11}B NMR Spectroscopy. Inorg. Chem. 1994, 33, 3811–3816. DOI: 10.1021/ic00095a026.

(4) Sandström, J. Dynamic NMR Spectroscopy; Academic Press: London, 1982.

(5) Vidal, F.; Gomezcoello, J.; Lalancette, R. A.; Jäkle, F. Lewis Pairs as Highly Tunable Dynamic Cross-Links in Transient Polymer Networks. *Journal of the American Chemical Society* **2019**, *141* (40), 15963–15971. DOI: 10.1021/jacs.9b07452. Published Online: Sep. 26, 2019.
